# The CYP4/20-HETE/GPR75 axis in the progression of metabolic dysfunction-associated steatosis liver disease (MASLD) to chronic liver disease

**DOI:** 10.3389/fphys.2024.1497297

**Published:** 2025-01-29

**Authors:** James P. Hardwick, Byoung-Joon Song, Paul Rote, Charles Leahy, Yoon Kwang Lee, Alexandra Rudi Wolf, Danielle Diegisser, Victor Garcia

**Affiliations:** ^1^ Department of Integrative Medical Sciences Liver Focus Group, Northeast Ohio Medical University, Rootstown, OH, United States; ^2^ Section of Molecular Pharmacology and Toxicology, National Institute on Alcohol Abuse and Alcoholism, Bethesda, MD, United States; ^3^ Department of Pharmacology, New York Medical College, Valhalla, NY, United States

**Keywords:** MASLD, *CYP4A11*, *GPR75*, 20-HETE, chronic liver disease

## Abstract

**Introduction:**

Metabolic-dysfunction-associated steatosis liver disease (MASLD) is a progressive liver disease from simple steatosis, steatohepatitis, fibrosis, cirrhosis, and hepatocellular carcinoma. Chronic liver diseases (CLDs) can lead to portal hypertension, which is a major cause of complications of cirrhosis. CLDs cause structural alterations across the liver through increased contents of extracellular matrix (ECM), driving dysfunction of liver sinusoidal endothelial cells (LSECs) alongside hepatic stellate cells (HSCs) and activated resident or infiltrating immune cells. Bioactive arachidonic metabolites have diverse roles in the progression of MASLD. Both secreted levels of 20-hydroxyeicosatetraenoic acid (20-HETE) and epoxyeicosatrienoic acid (EET) are elevated in patients with liver cirrhosis.

**Methods:**

CLD samples were evaluated for changes in free fatty acids (FFA), cholesterol, bilirubin, bile acid, reactive oxygen species (ROD), lipid peroxidation, myeloperoxidase activity and hydroxyproline levels to evaluate the degrees of liver damage and fibrosis. To address the role of the CYP4/20-HETE/GPR75 axis, we measured the amount and the synthesis of 20-HETE in patients with CLD, specifically during the progression of MASLD. Additionally, we evaluated gene expression and protein levels of GPR75, a high-affinity receptor for 20-HETE across CLD patient samples.

**Results:**

We observed an increase in 20-HETE levels and synthesis during the progression of MASLD. Increased synthesis of 20-HETE correlated with the expression of *CYP4A11* genes but not CYP4F2. These results were confirmed by increased P4504A11 protein levels and decreased P4504F2 protein levels during the development and progression of MASLD. The gene expression and protein levels of GPR75, the major receptor for 20-HETE, increased in the progression of MASLD. Interestingly, the *CYP4A11* and *GPR75* mRNA levels increased in steatohepatitis but dramatically dropped in cirrhosis and then increased in patients with HCC. Also, protein levels of P4504A11 and GPR75 mirrored their mRNA levels.

**Discussion:**

These results indicate that the *CYP4A11* and subsequent *GPR75* genes are coordinately regulated in the progression of MASLD and may have multiple roles, including 20-HETE activation of peroxisome proliferator-activated receptor α (PPARα) in steatosis and GPR75 in CLD through either increased cell proliferation or vasoconstriction in portal hypertension during cirrhosis. The abrupt reduction in *CYP4A11* and *GPR75* in patients with cirrhosis may also be due to increased 20-HETE, serving as a feedback mechanism via *GPR75*, leading to reduced *CYP4A11* and *GPR75* gene expression. This work illustrates key correlations associated with the CYP4/20-HETE/GPR75 axis and the progression of liver disease in humans.

## 1 Background introduction

Metabolic-dysfunction-associated steatotic liver disease (MASLD) is a progressive disease stemming from steatosis, followed by steatohepatitis (MASH) and chronic liver disease (CLD), including cirrhosis and hepatocellular carcinoma. The overall prevalence of non-alcoholic fatty liver disease (NAFLD), presently referred to as MASLD, due to the intimate association of the disease alongside various metabolomics attributes and factors, worldwide is estimated to be 32.4% and has steadily increased over the last 3 decades (Riazi et al., 2022). Chronic liver disease (CLD) accounts for two million deaths annually and is responsible for 4% of all deaths worldwide. Deaths are attributable primarily to complications of cirrhosis and hepatocellular carcinoma. The most common causes of cirrhosis worldwide are related to viral hepatitis, alcohol drinking, and MASLD (Devarbhavi et al., 2023). Cirrhosis is an important driver of morbidity and mortality among patients with CLD. Cirrhosis can progress, leading to hepatocellular carcinoma (HCC) and hepatic decompensation, including portal hypertension, ascites, hepatic encephalopathy, and variceal bleeding, and is associated with 2.4% of global deaths in 2019 (Huang et al., 2023). Portal hypertension in CLD is the major cause of morbidity and mortality in patients with cirrhosis. Portal hypertension is initiated by increased intrahepatic vascular resistance and a hyperdynamic circulatory state. A high cardiac output characterizes the latter, as well as increased total blood volume and splanchnic vasodilatation, resulting in increased mesenteric blood flow and cirrhotic portal hypertension (Gunarathne et al., 2020).

Eicosanoid production is increased in patients with MASLD and CLD. The vasodilatory eicosanoids, including epoxyeicosatrienoic acid (EET) and vasoconstrictive 20-hydroxyeicosatetraenoic acid (20-HETE), are elevated in the urine of patients with CLD (Sacerdoti et al., 1997). *CYP4* ω-hydroxylase is responsible for the metabolism of arachidonic acid (AA) to pro-inflammatory 20-HETE. CYP4 ω-hydroxylase is also responsible for the metabolism of leukotrienes (LTs), prostaglandins (PGs), and EETs. A lipidomic study has identified eleven eicosanoids (8−HETE, 20−HETE, 11,12−DiHETrE, 14,15−DiHETrE, 11−keto-TXB2, LTE, and 12-HHT) that discriminated HCC patients from those with decompensated cirrhosis and acute on chronic liver disease (ACLD) (Lopez-Vicario et al., 2020). Using the lipidomics approach, a NASH score of 9- and 13-HODEs and 9- and 13-oxoODEs, products of free radical-mediated oxidation of linoleic acid (LA), was significantly elevated in patients with non-alcoholic steatohepatitis (NASH) compared to patients with steatosis (Feldstein et al., 2010). Very little information is available about the role of 20-HETE in the CLD. However, 20-HETE participates in the regulation of liver metabolic activity and hemodynamics (Sacerdoti et al., 2015). In fact, 20- HETE is a potent activator of PPARα and may exert essential functions in lipid homeostasis and in controlling fat-dependent energy supply and metabolism. In addition, it is an important inflammatory mediator and may have a critical role in inflammatory diseases (Antoun et al., 2008).

Recently, the pairing of the G-protein coupled receptor (GPCR.) GPR75 and 20- HETE have been identified (Garcia et al., 2017; Cardenas et al., 2023; Tunctan et al., 2022). Recent studies suggest that 20-HETE, through GPR75, triggers signaling pathways, including PI3K/Akt and RAS/MAPK, leading to cell proliferation, migration, and apoptosis, depending on the cellular context (Cardenas et al., 2023; Tunctan et al., 2022; Pascale et al., 2023). Patients with truncating variants in GPR75 exhibit lower body-mass index BMI and a 54% lower odds ratio toward obesity in the heterozygous state. Moreover, GPR75 variants are linked to a reduced risk of developing hepatic steatosis (Akbari et al., 2021; Leeson-Payne et al., 2024). Interestingly, *CYP4A14* gene-deficient mice fed a Westen-style high-fat diet or a methionine and choline-deficient (MCD) diet exhibited decreased liver lipid accumulation with reduced hepatic inflammation and fibrosis (Zhang et al., 2017). These results suggest that 20-HETE and GPR75 play a pivotal role in developing CLD and MASLD. While 20-HETE and prostaglandins (PGs) are excreted at similar rates in normal subjects, it was reported that excretion rates of 20- HETE were several-fold higher than those of PGs and Thromboxane B_2_ (TXB_2_) in patients with cirrhosis (Sacerdoti et al., 1997). Additionally, 20-HETE can interfere with liver nitric oxide synthesis by activating the renin-angiotensin system (RAS), including changes to endothelial angiotensin-converting enzyme (ACE) expression. The role of 20-HETE still needs to be determined in liver physiology, although it represents 50%–75% of CYP-dependent AA metabolites in this organ. Therefore, future studies investigating the physiological and pathological roles of hepatic 20-HETE will be critical, as important in the functional relationship of the *CYP4* and GPR75 genes in the progression of MASLD and the role in portal hypertension in cirrhosis and CLD.

**FIGURE 1:**
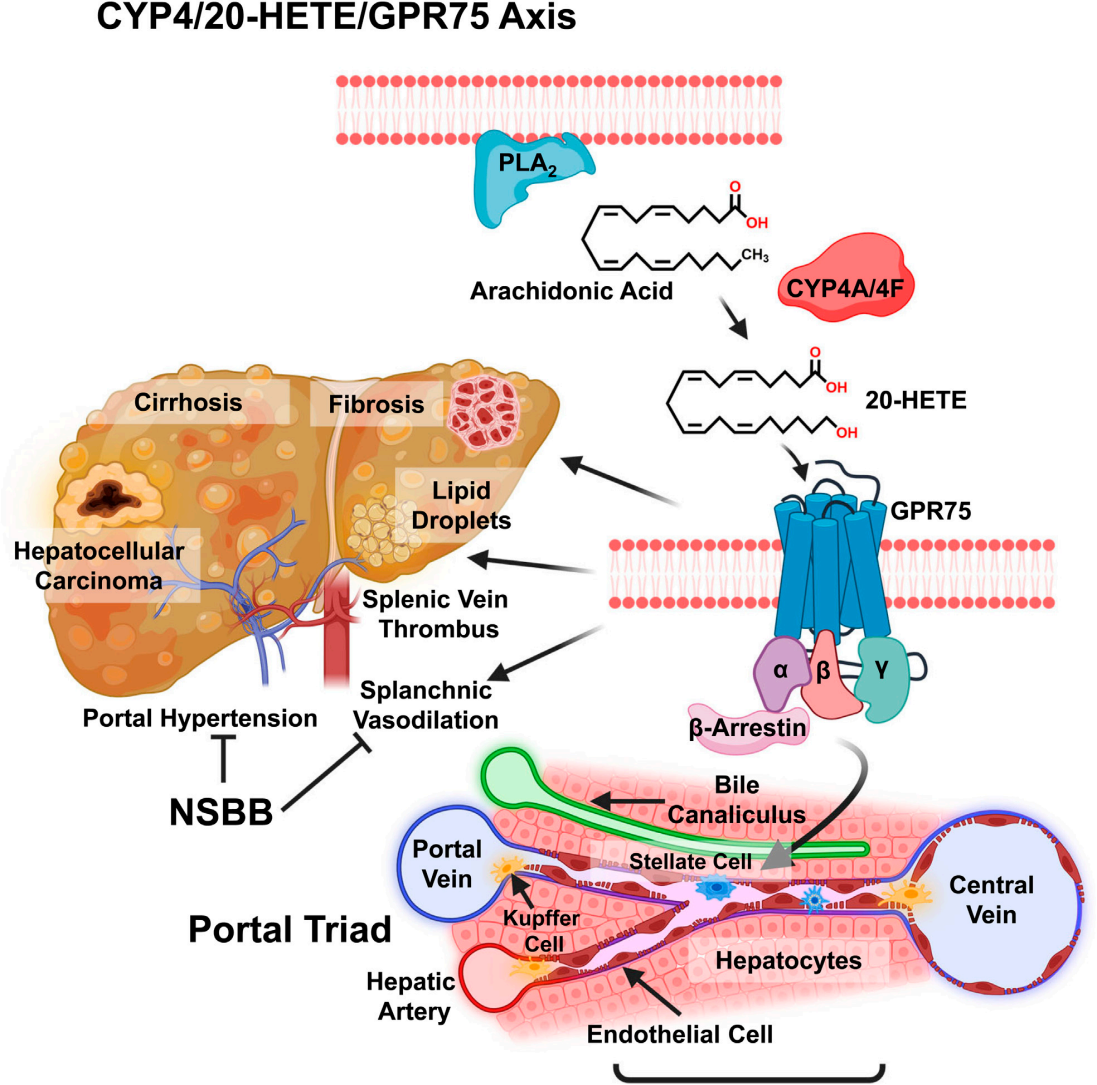
Graphic view on the role of CYP4 arachidonic acid ω-hydroxylase and the 20-HETE receptor GPR75 in chronic liver disease (CLD). The liver is in different stages of CLD, and various factors are shown, from the accumulation of lipid droplets in steatosis to fibrosis, cirrhosis, and hepatocellular carcinoma, with the significant complication of portal hypertension seen in the progression of MASLD. Metabolism of arachidonic acid (AA) by CYP4A/4F ω-hydroxylase to 20-HETE, which activates the GPR75 receptor, eliciting different unknown responses by liver Kupffer cells, hepatocytes, endothelial cells, bile canaliculi cells, and activation of stellate cells in hepatic fibrosis. Non-selective β-blockers (NSBB) inhibit vasoconstriction of portal vein vasoconstriction and portal hypertension.

Despite our understanding of the mechanisms involved in the development and progression of MASLD, there are currently no approved pharmacological treatments for MASLD and its advanced forms in patients with and without T2DM. The only approved therapy for MASLD/MASH is Rezdiffra, which was based on the resolution of NASH and improved fibrosis in about a quarter of the patients treated in a large international trial (MAESTRO MASH, NCT03900429) (Alshehade, 2024). This drug works by revving up the thyroid hormone pathway in the liver to increase liver fat metabolism. In CLD, initial pharmacological manipulation of cirrhotic portal hypertension targets both the splanchnic and hepatic vascular beds targeted by the classical RAS inhibitors that are expected to decrease intrahepatic vascular tone by reducing extracellular matrix (ECM) deposition and the activity of endothelial and stellate contractile cells. However, these drugs produced significant off-target effects such as systemic hypotension and renal failure. The current pharmacological mainstay in clinical practice is non-selective beta-blockers (NSBBs). These NBs reduce cardiac output and splanchnic vasodilation, but most patients do not achieve an optimal therapeutic response. Although statins, used alone or in combination with NSBBs, have been shown to improve portal pressure, they did not improve mortality in cirrhotic patients (Pfi et al., 2024). Despite significant advances in understanding the pathophysiology of MASLD and portal hypertension in CLD, treatment options for these conditions are limited. Understanding the role of the CYP4/20-HETE/GPR75 axis in the progression of MASLD and the effective management of portal hypertension in CLD and cirrhosis may provide new therapeutic avenues to treat these progressive liver diseases (Figure 1).

## 2 Methods

### 2.1 Human tissues

Human snap-frozen liver samples were obtained from the Cooperative Human Tissue Network and the Liver Tissue Distribution Center (LTDC) of the University of Minnesota, Project Number 276201200017C-3-0-1. Northeast Ohio Medical University IRB approved using human tissues and cells with protocol 01-043. Twenty-eight liver samples from normal, steatosis, steatohepatitis, cirrhosis, and hepatocellular carcinoma from patients 40–75 years old and at each stage of MASLD, totaling 140 samples, equal numbers of liver samples from males and females were analyzed. All samples were histologically graded by a pathologist into the NAFLD activity score (NAS) ranging from 0 to 8 and calculated by the sum of scores of steatosis (0–3), lobular inflammation (0–3), and hepatocyte ballooning (0–2). We characterized liver samples as having steatosis with a NAS below four and livers with steatohepatitis having an activity score of 4 and above. Liver samples with NAS scores above four were also assigned a fibrosis score of F0-F4. Steatohepatitis liver samples had F1 to F3, and patients with cirrhosis displayed bridging fibrosis and regenerative nodules with fibrosis scores of F3 and F4. Hepatocellular carcinoma was graded according to the WHO’s three-stage system. There are well-differentiated (G1), moderately differentiated (G2), and poorly differentiated (G3) HCCs.

### 2.2 Processing of human tissues

One Gram of snap-frozen liver was homogenized in 25 mM Tris-pH 7.5, 0.25 M sucrose, 1 mM EDTA, and 2 mM MgCl2 containing a protease inhibitor cocktail (Sigma P8340), 1 μM butylated hydroxytoluene (BHT), 1 µM diethylenetraiaminepentaacetic acid (DTPA) briefly with polytron to break tissue, and then 10 strokes with a Potter-Elvehjem homogenizer. The homogenate was centrifuged at 300 g to pellet debris and then filtered through two layers of gauze. The homogenate was centrifuged sequentially at ×1,000 g to pellet nuclei and ×10,000 g to pellet mitochondria. The supernatant was centrifuged at ×100,000 g for 1 h to pellet microsomes suspended in 10 mM Tris-HCL, 25% (v/v) glycerol, 1 mM EDTA, and protease inhibitors. The total homogenate and microsomes were stored at −80° until further usage.

### 2.3 Biochemical assays

The Wako HR series NEFA-HR kit and the Wako Cholesterol E assay kit were used to determine the amounts of non-esterified fatty acid (NEFA) and total cholesterol in liver extracts, respectively. Total bile acids (TBA) were determined using a colorimetric Assay Kit from BioVision. Bilirubin (Total and Direct) Colorimetric Assay Kit and Hydroxyproline Colorimetric Assay Kit were from BioVision, Inc. to measure the levels of bilirubin and hydroxyproline, respectively. The Lipid Hydroperoxide (LPO) Assay Kit and Neutrophil Myeloperoxidase Activity Assay Kit were from Cayman Biochemicals to determine LPO and myeloperoxidase activity levels, respectively. Reactive oxygen species (ROS) were determined using an Amplex™ Red hydrogen peroxide/Peroxidase Assay Kit (Thermo- Fisher).

### 2.4 RNA isolation and cDNA synthesis

RNA was isolated from 100 mg of liver tissue dissolved in 1 mL RNAzol^®^ RT from the Molecular Research Center (MRC). RNA was suspended in 200 µL of water and 200 µL of 5 M LiCl and kept at −20°C for 1 h before centrifugation at 17,000 x g for 20 min. The RNA pellet was washed in 75% ethanol and suspended in 200 µL of RNase-free water. Twenty ug of total RNA was reverse transcribed with RocketScript reverse transcriptase for 90 min at 55°C. The 20 µL cDNA reaction was increased to 400 µL of 1X buffer 3 (New England Biolabs) and then treated with 100 units of RNase If (New England Biolabs, Inc.) for 1 h at 37°C. The amount of single-strand (ss) cDNA and double-stranded (ds) DNA was determined using Quantifluor detection kits from Promega Corporation. The amount of cDNA was determined by the amount of ssDNA minus the amount of dsDNA expressed as a nanogram of cDNA per µL.

### 2.5 Absolute PCR quantitation of mRNA levels

Linear regression of efficiency (LRE) introduced a new paradigm for real-time qPCR that enables large-scale absolute quantification by eliminating the need for standard curves by using lambda DNA as a universal quantitative standard (Rutledge et al., 2010). The LRE program can be downloaded from https://code.google.com/archive/p/lreqpcr/downloads. Standard reference Lambda DNA reference 2372a was obtained from the National Institute of Standards and Technology (NIST) and diluted to 50 fg (fg) and 100 fg. One hundred femtograms equals 1876 molecules and has a Ct value of 26. Quantitation of mRNA was performed in white 96-well PCR plates with 100 fg and 200 fg of lambda DNA standards. Touchdown (TD.) PCR offers a simple and rapid way to optimize PCRs, increasing specificity, sensitivity, and yield without requiring lengthy optimizations or redesigning primers (Korbie and Mattick, 2008). PCR was performed in an ABI. Fast 7,500 unit at 95°C for 2 min, followed by one cycle at 66°C, 64°C, 62°C, and then 55 cycles at 60°C with SYBR Green and Luna PCR mix (New England Biolab, Inc.). PCR results were analyzed with the LRE software program and expressed as the number of molecules of mRNA per ng cDNA. Results from two sets of primer pairs for each gene were compared for accuracy (Supplementary Table S1).

### 2.6 Western immunoblot analysis

Protein levels were quantitated after separation by gel electrophoresis of either total or microsome extracts in a 10% polyacrylamide gel blotted to an Immobilon-P PVDF membrane. Membranes were blocked in 5% fat-free dry milk in Tris-buffer saline and then incubated with peptide-specific antibodies to P4504A11, P4504F2, P4504A22, GPR75, and β-actin (Supplementary Table S2). The rabbit primary antibodies were detected by incubating blots with anti-rabbit conjugated biotin and streptavidin-conjugated alkaline phosphatase or horseradish peroxidase. All five blots of normal steatosis, NASH, cirrhosis, and HCC were treated simultaneously and under the same conditions, thus allowing for accurate comparison among different human samples. The total protein of each sample was determined by running samples in 4% stacking gels containing 0.5% 2,2,2-trichloroethane (Chopra et al., 2019). Gels were incubated in 20% (W/V) trichloroacetic acid for 30 min. UV exposure activated the gel at 310 nm for 3 min, and then a single band in each well was detected by fluorescence emission at 310 nm excitation and emission at 450 nm. We also normalized each lane to β-actin by first incubating and developing the blot with the target antibody and then re-incubating the blot with an anti-actin antibody. Therefore, we have the target protein and actin picture on the same blot. The amount of antibody-detected protein was reported relative to either total protein or β-actin used as a loading control. Using either β-actin or total protein to normalize the target protein gave similar results.

### 2.7 GC-MS detection of 20-HETE

One mg of total extract in 1 mL of Tris-HCL, 0.15 M NaCl, was made acidic with 100 µL of 6 N HCl, then extracted 3x with 1 mL of ethyl acetate containing 10 µL of antioxidant cocktail (2 mg/mL EDTA, 0.2 mg/mL butylated hydroxytoluene, 1 mg/mL triphenylphosphine, 1 mg/mL indomethacin in ethanol/methanol/water (1/1/2), and either 2 ng of 20-HETE-d6 or 17-hydroxy heptadecanoic acid (17-OHC17). The extracted lipids were dried under nitrogen. To determine the rate of 20-HETE formation, 40 µM arachidonic acid was added to siliconized tubes containing 1 mg of microsome protein, 0.05 µM indomethacin, 50 mM NaHPO4, pH 7.4, 5 mM isocitrate and 0.05 units of isocitrate dehydrogenase on ice for 30 min. The 1 mL reaction was incubated for 5 min at 37°C, then initiated by adding 1 mM NADPH. The reaction was incubated for 30 min, and 10 µL of antioxidant was added with either 2 ng 20-HETE-d6 or 17OHC17. The microsome reaction was extracted with acidic ethyl acetate pH 3.5 three times and dried under Nitrogen. The lipids were suspended in 100 µL of acidic ethyl acetate and purified by solid phase extraction (SPE). A 60 mg OasisPrime HLB cartridge was pre-conditioned with a 3 mL SPE solution (water/methanol/acetic acid, 950/49/1) and a 10 µL antioxidant cocktail. The 100 µL samples were diluted to 1 mL with SPE and then applied to the cartridge by gravity flow. The column was washed with 3 mL of SPE and left to dry. Long-chain fatty acids and oxylipins were eluted with 1.5 mL of ethyl acetate, dried, and suspended in 100 µL ethyl acetate. Samples were derivatized for GC-MS analysis with Trimethylsilyl (TMS) and N, O-bis(trimethyl-silyl)-trifluoroacetamide (BSTFA) reagents or the formation of tert- butyl dimethylsilyl (TBDMS) derivatives with N-tert-butyldimethylsilyl-N- methyltrifluoroacetamide (MTBSTFA). Fifty µL of BSTFA or MTBSTFA was added to samples and heated to 80°C for 60 min. Samples were analyzed using an Agilent 5973N- MSD equipped with an Agilent 6890 GS system (Santa Clara, CA) on a TRACE™ TR- FFAP GC Columns, 0.25 μm, 0.25 mm ID, 15 m length. The run was performed under an optimized temperature program of 90°C initial, hold of 5 min, increase by 5°C per min to 130°C, and an increase by 40°C per min to 240°C. The split ratio was varied for different samples with a helium flow of 1 mL per min and a mass spectrometer operating in electron impact (EI) mode. The analytes were quantified in the timed selected ion monitoring (SIM) mode using the target ion and verified by confirmative ions. Ion chromatograms were quantified using the Auto-Integrate function with Agilent ChemStation software.

### 2.8 Statistical analyses

All data in this study are denoted as mean +SEM. To test the statistical significance between the five groups, One-way analysis of variance (ANOVA) was performed, followed by the Bonferroni-Dunn *post hoc* correction using GraphPad Prism software version 10.2.3 (San Diego, CA, USA). For all analyses, *p* < 0.05 was considered statistically significant.

## 3 Results

### 3.1 Human liver tissue clinical analysis

A total of 28 human liver samples were obtained at each stage in the progression of MASLD (normal, steatosis, NASH, cirrhosis, and hepatocellular carcinoma (HCC)) from the Cooperative Human Tissue Network (CHTN) or Liver Tissue Distribution System (LTDS) at the University of Minnesota. A pathologist histologically graded all samples into the different stages of MASLD.

**FIGURE 2:**
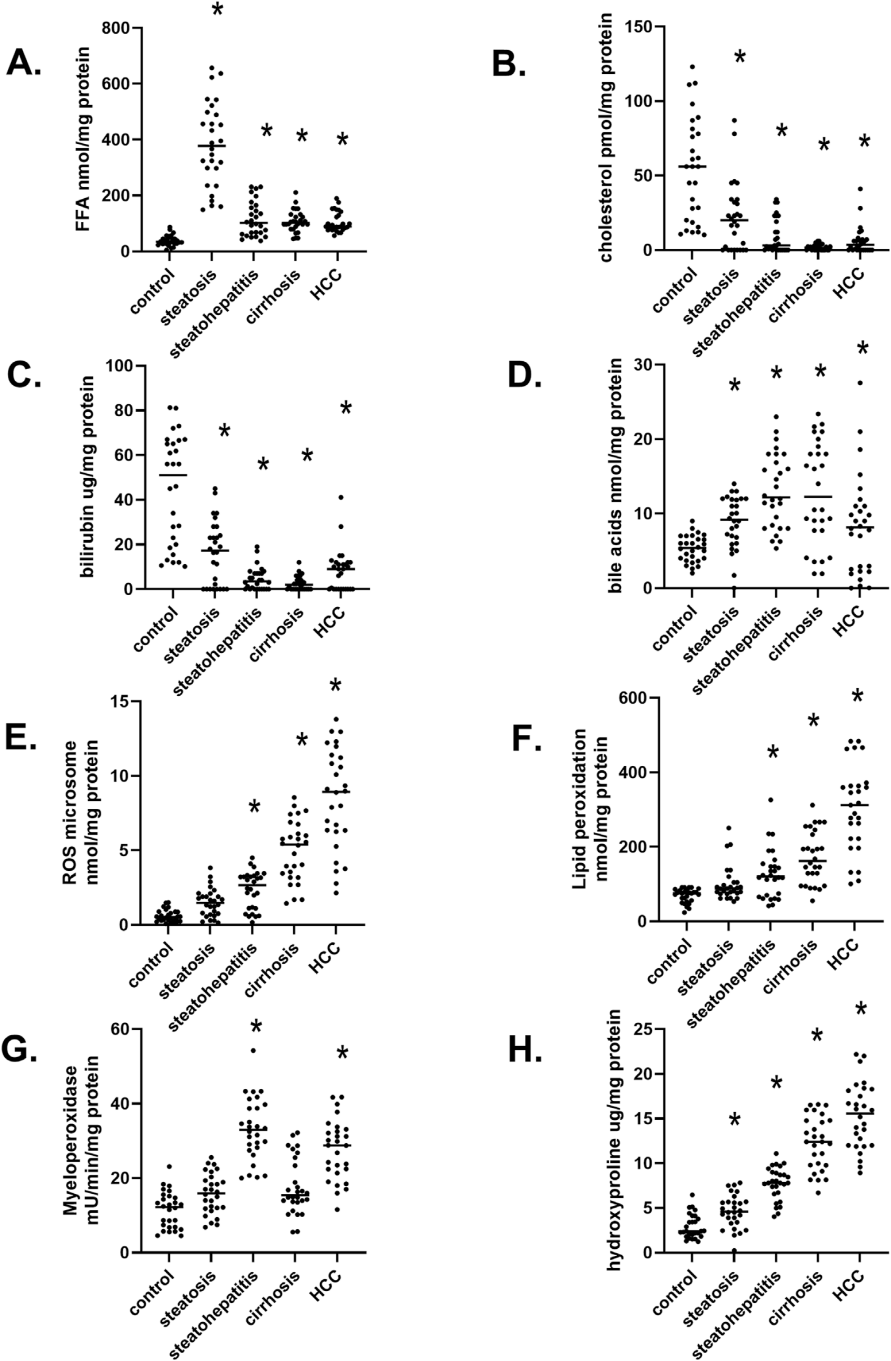
Biochemical markers in twenty-eight liver samples at different stages of MASLD progression **(A)** Free fatty acid (FFA) levels at various stages of liver diseases. **(B)** Hepatic Cholesterol levels, **(C)** The concentration of liver bilirubin, **(D)** Total bile acid amount at different stages of MASLD, **(E)** Reactive oxygen species (ROS) in the liver, **(F)** The level of lipid peroxidation product, **(G)** The activity of myeloperoxidase in MASLD progression, **(H)** The degree of liver fibrosis determined by hydroxyproline levels. n = 28 per group, 140 total patient samples. Statistically significant changes relative to control normal livers are indicated by *p < 0.05.

A significant increase in liver-free fatty acid (FFA) was observed in hepatic steatosis. Still, FFA levels decreased during the progression of MASLD (Figure 2A). In contrast, liver cholesterol levels continually dropped in the progression of MASLD (Figure 2B), most likely due to increased transport/excretion into the blood as VLDL and LDL. Serum bilirubin levels are a marker of the end-stage liver disease (MELD) scoring model (Singal and Kamath, 2013). Interestingly, liver bilirubin levels also fell in the progression of MASLD, which can reflect changes in increased transport into blood (Figure 2C) (Guerra Ruiz et al., 2021). In contrast to decreased liver cholesterol and bilirubin levels, there is a continual increase in bile acids in the progression of MASLD. (Figure 2D), a diagnostic marker used in the Child-Pugh scoring system that rates the severity of long-term liver disease and cholestasis (Peng et al., 2021; Xie et al., 2017). The differential levels of bile acids and bilirubin during the progression of MASLD may be due to the downregulation of the bile salt exchange protein (BSEP) in CLD with an upregulation of multi-drug resistant transport protein 2 (MRP2/ABCC2). It is known that MRP2 transport of estradiol-17β-D-glucuronide inhibits BSEP (Liu et al., 2024). During the progression of MASLD, we observed a significant and continued increase in the levels of reactive oxygen species (ROS) (Figure 2E) and lipid peroxidation (Figure 2F) (Jakubek et al., 2024). Studies in patients with MASLD showed correlations between elevated oxidative stress indicators, diminished serum antioxidant levels, and the progression of MASLD (Masarone et al., 2018). We also observed increased immune cell myeloperoxidase (MPO) activity in liver samples with steatohepatitis and hepatocellular carcinoma (Figure 2G). Elevated MPO activity in the liver and plasma has been associated with metabolic syndrome and cardiovascular and liver disease in MASLD (Jakubek et al., 2024). During the progression of MASLD, we observed a continued increase in hydroxyproline levels (Figure 2H), indicating increased liver fibrosis, one of the most critical features of wounded tissues. The progression of fibrosis depends mainly on the imbalance between the rate of formation and degradation of collagen, which is associated with many metabolic and biochemical abnormalities. The hydroxyproline level in liver tissues, serum, and urine represents liver fibrogenesis rates and progression (Karsdal et al., 2020). These clinically relevant data indicate that our liver samples illustrate the dynamic and subsequential stages in the progression of MASLD.

### 3.2 Arachidonic acid metabolism and 20-HETE level in MASLD

**FIGURE 3:**
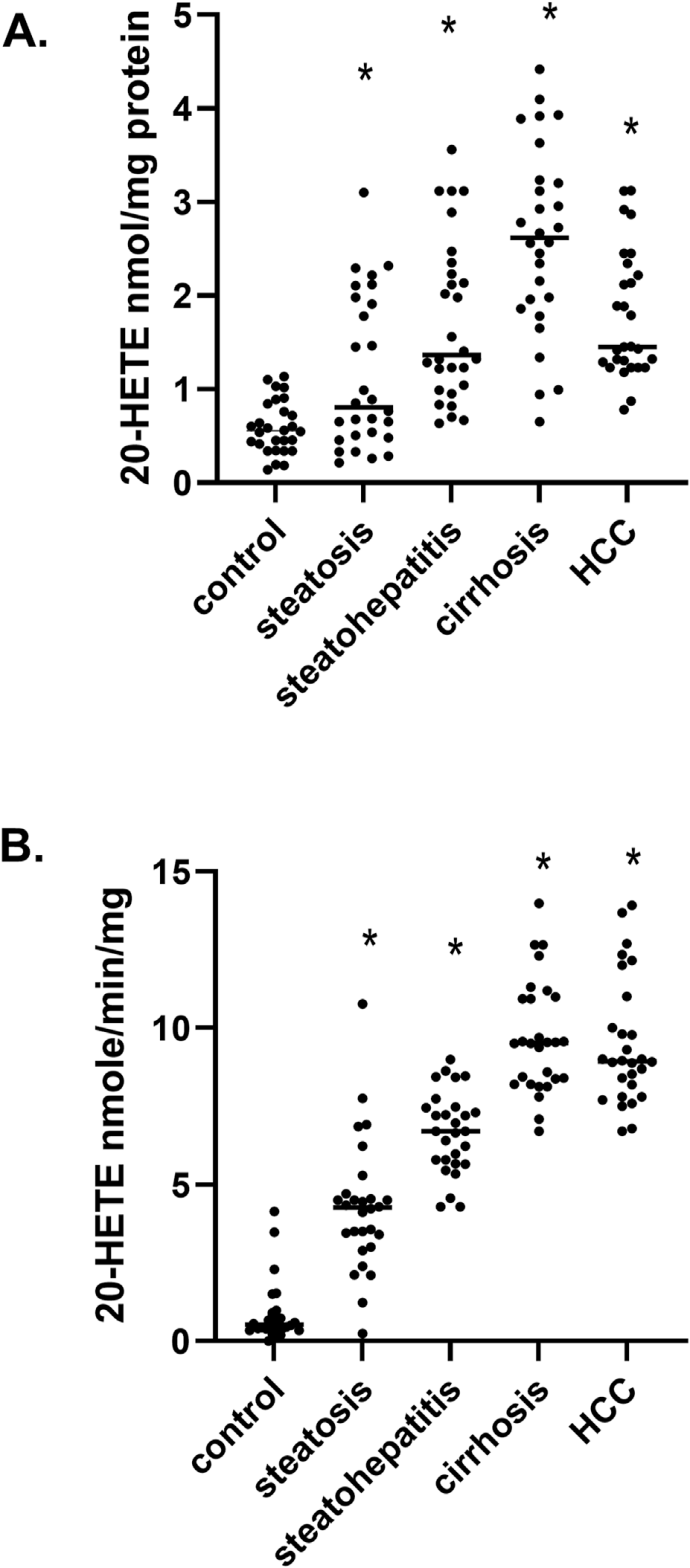
20-HETE levels and its synthesis rates in MASLD **(A)** The level of 20-HETE in hepatic tissues at different stages of MASLD, **(B)** the synthesis rates of 20-HETE in liver microsomes at different stages of MASLD n = 28 per group, 140 total patient samples. Statistical significance compared to normal control liver *p < 0.05.

Via gas-chromatography mass spectrometry, we measured the levels of 20- hydroxyeicosatetraenoic acid (20-HETE) in liver samples and observed a significant and continued increase in 20-HETE levels from steatosis up to cirrhosis and subsequently a minor reduction in HCC samples (Figure 3A). To determine if the increase in 20-HETE levels was due to increased ω-hydroxylation of arachidonic acid (AA), we measured the synthesis rates of 20-HETE in microsomes at different stages of MASLD (Figure 3B). We observed a continual increase in the rate of 20-HETE formation during the progression of MASLD, and this elevation is consistent with changes in 20-HETE levels (Figure 3A). These results suggest members of the cytochrome P450 fatty acid ω-hydroxylase gene family (*CYP4*) may be responsible for the metabolic conversion of AA to 20-HETE.

### 3.3 Quantitative analysis of *CYP4* gene expression and protein levels in the progression of MASLD

#### 3.3.1 Analysis of absolute mRNA and quantitation of total protein

To understand the function of a particular gene, it is necessary to know the number of RNA transcripts arising from this gene under different physiological conditions, cell types, tissues, or development stages. However, the transcription of reference or housekeeping genes and the transcriptome vary depending on cell type, organism, tissue, and pathophysiological condition (Baker et al., 2005). Quantitative real-time reverse transcription polymerase chain reaction: normalization to rRNA or single housekeeping genes could be inappropriate for analyzing human biopsied tissue. Inter-individual variation in gene expression of reference or housekeeping genes thus makes it challenging to analyze the target gene between individuals. To overcome this inconsistency in assessing target mRNA levels in different tissues and individuals, we used absolute quantitative PCR to determine target mRNA levels expressed as the number of molecules per nanogram of cDNA in normal liver and individuals with steatosis, steatohepatitis, cirrhosis, and hepatocellular carcinoma. Our results of molecules of mRNA per ng cDNA agree with the number of transcripts per million (TPM) of genes in RNA-seq datasets for fatty liver disease. GSE135251 (n = 256), GSE114564 (n = 118), GSE174478 (n = 74), GSE 167823 (n = 100), GSE 162694 (n = 144), GSE 124535 (n = 72), GSE 114564 (n = 144), and GSE63018 (n = 36).

Similarly, normalizing target protein levels between individuals and different disease states presents a challenge to identifying housekeeping genes that remain stable in other individuals and disease states. Thus, we chose to normalize our samples to total protein because it was shown that both β-actin and glyceraldehyde phosphate dehydrogenase (GAPDH) showed a 20% coefficient of variation (CV) in the human samples and a 1.45-2.88 fold change in β-actin in HCC tissue compared to matched normal tissue (Hu et al., 2016). We normalized total protein pixel intensity between normal, steatosis, steatohepatitis, cirrhosis, and HCC liver samples, and this approach provided a picture of a change in the target protein during the progression of MASLD. We also sequentially developed blots to target protein and β-actin. Therefore, on the same blot, we can normalize the target protein to the level of β-actin in each lane.

#### 3.3.2 CYP4A11

**FIGURE 4:**
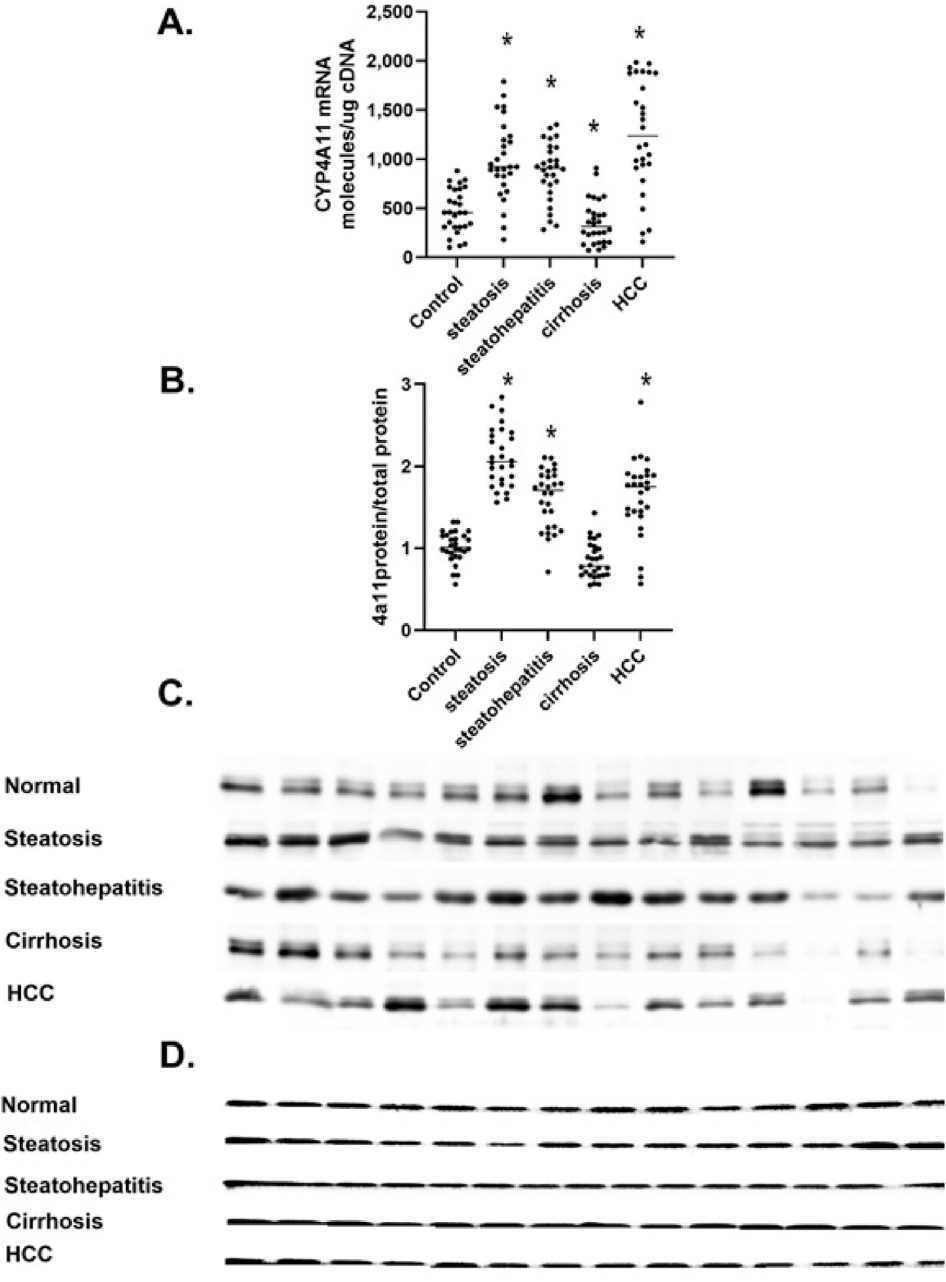
The level of *CYP4A11* mRNA and protein in MASLD. **(A)** Changes in *CYP4A11* mRNA as mRNA molecules per nanogram cDNA, **(B)** The level of P4504A11 protein, **(C)** Western immunoblot of P4504A11 protein at different stages of MASLD, **(D)** Total protein stain of proteins separated in a 4% stacked polyacrylamide gel at different stages of MASLD n = 28 per group, 14 representative immunoblot samples per group. Statistical significance compared to control liver *p < 0.05.

The *CYP4A11* and *CYP4F2* P450s are the major microsomal enzymes responsible for the metabolism of AA to 20-HETE in the human liver. We measured the levels of CYP4A11 mRNA at different stages of MASLD progression (Figure 4A). *CYP4A11* mRNA levels significantly increased in steatosis and steatohepatitis. Still, they dramatically decreased in cirrhosis and then increased in HCC. This dynamic pattern was confirmed with two *CYP4A11* primer sets (Supplementary Table S1). Also, the number of molecules of *CYP4A11* mRNA per ng of cDNA agreed well with the TPM of several datasets (GSE114564, GSE135251) (Leahy et al., 2024). This data suggests that absolute quantitative PCR of mRNA molecules normalized to cDNA levels is a valid method to determine the target mRNA levels in different tissues and individuals. To confirm our *CYP4A11* mRNA results, we analyzed the levels of P450 4A11 by Western immunoblot analysis with peptide-specific antibodies (Figure 4B; Supplementary Table S2).

*CYP4A11* protein levels increased in steatosis and steatohepatitis but dramatically decreased in cirrhosis and then increased in HCC (Figure 4B). The increase in P4504A11 protein levels in steatosis, steatohepatitis, and HCC cirrhosis is consistent with the altered patterns of *CYP4A11* mRNA results (Figures 4A, B). The dramatic decline in *CYP4A11* mRNA and protein (Figures 4B, C) in cirrhosis has been confirmed with multiple *CYP4A11* primer sets and two different P4504A11 peptide-specific antibodies compared to β-actin used as a loading control (Figure 4D).

#### 3.3.3 CYP4A22

**FIGURE 5:**
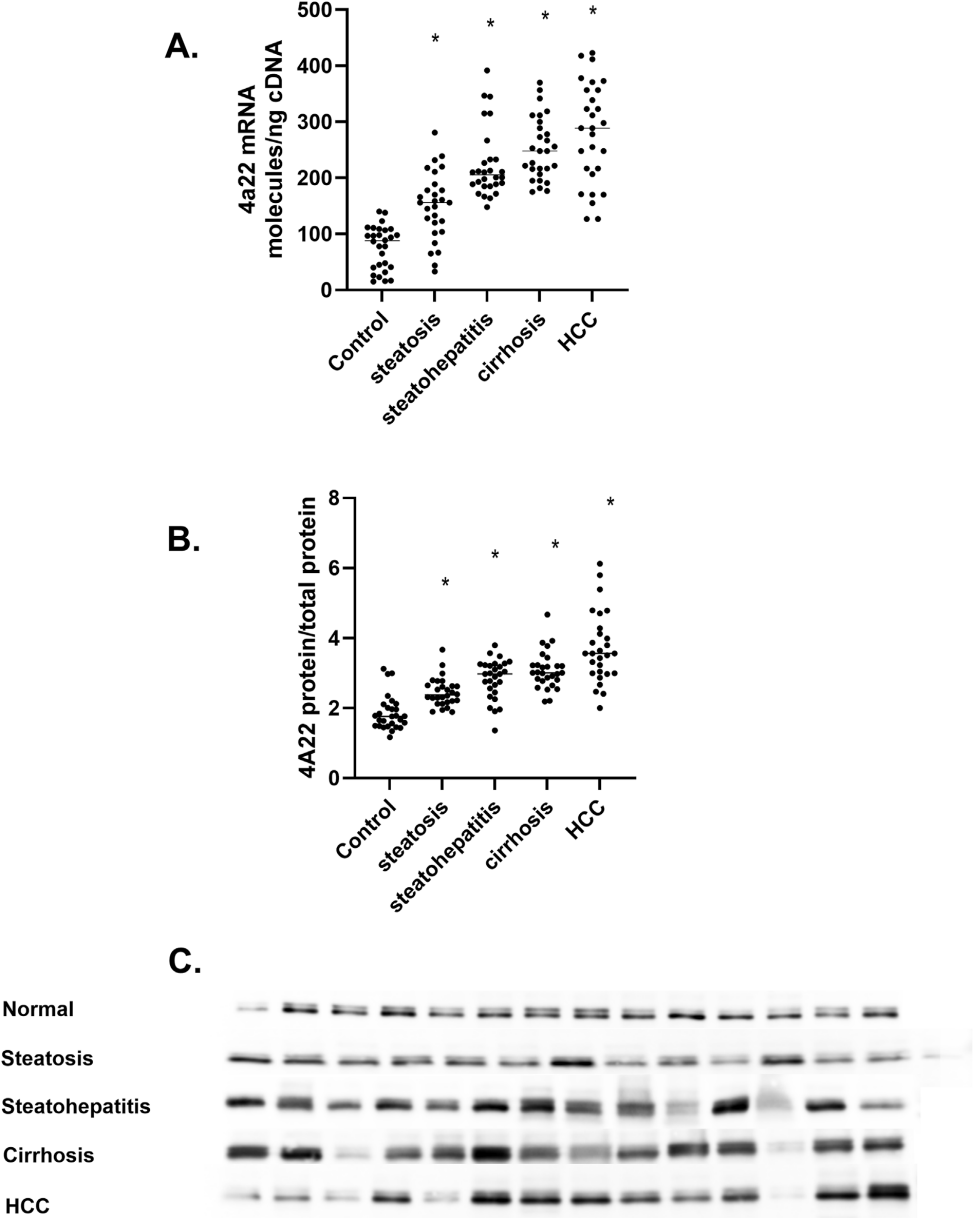
The amount of *CYP4A22* mRNA and protein in the progression of MASLD **(A)** *CYP4A22* mRNA levels as mRNA molecules per nanogram cDNA, **(B)** P4504A22 protein level at different stages of MASLD, **(C)** Western immunoblot of P4504A22 protein in different stages of MASLD n = 28 per group, 14 representative immunoblot samples per group. Statistical significance compared to control liver *p < 0.05.

*CYP4A22* is an orthologue to *CYP4A11* that catalyzes the ω-hydroxylation of lauric acid and myristic acid but not AA (Durairaj et al., 2019). Analysis of *CYP4A22* mRNA levels in the human liver samples shows a five-fold less quantity than those of *CYP4A11* mRNA, and these results agree with RNA-seq database analysis. The increase in *CYP4A22* mRNA was observed during the progression of MASLD (Figure 5A). A significant increase in P4504A22 protein levels (Figure 5C) in MASLD is confirmed. Unlike *CYP4A11* mRNA and P4504A11 protein that dramatically decrease in liver cirrhosis patients, *CYP4A22* mRNA and P4504A22 protein levels increase (Figures 5A–C). Recently, the *CYP4A22* gene has been implicated in vitamin D-dependent rickets through its ability to function as a 25-hydroxylase for vitamin D3 (Duan et al., 2024). Together, these results implicate members of the human CYP4 family involved in fatty acid metabolism and cholesterol and bile acid metabolism (Hardwick, 2015; Osborne et al., 2022).

#### 3.3.4 CYP4F2a

**FIGURE 6:**
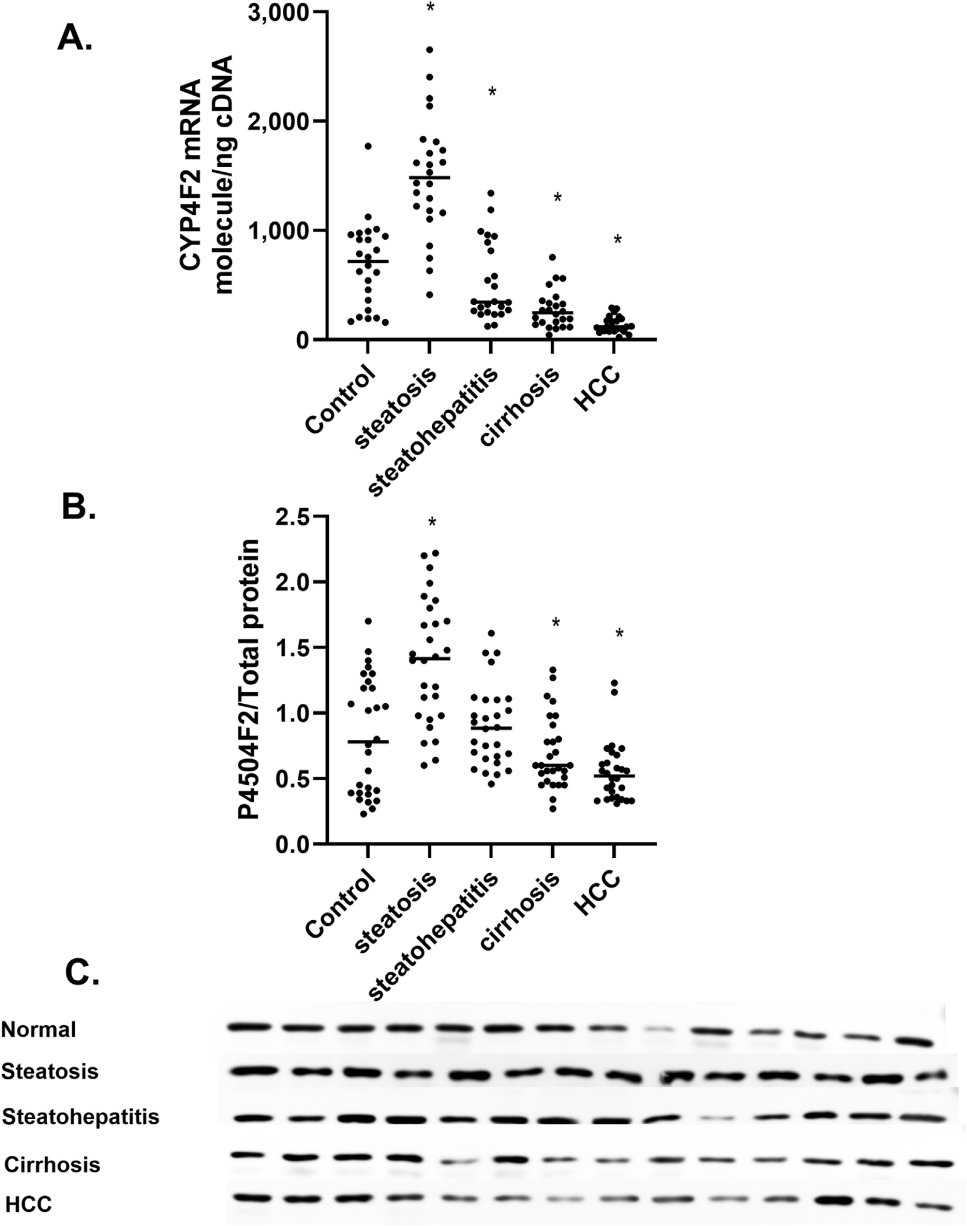
The amount of *CYP4F2* mRNA and protein at different stages of MASLD **(A)** *CYP4F2* mRNA expressed as mRNA molecules per nanogram cDNA, **(B)** Amount of P4504F2 protein at different stages of MASLD, **(C)** Immunoblot analysis of P4504F2 protein at different stages of MASLD n = 28 per group, 14 representative immunoblot samples per group. Statistical significance compared to control liver *p < 0.05.

The *CYP4F2a* gene is the primary P450 responsible for the formation of 20-HETE in humans, with a Kcat 14-fold higher than P4504A11 and a Km of 24 µM compared to 228 for P4504A11 (Leahy et al., 2024). The CYP4F2 P450 metabolizes several eicosanoids, including pro-inflammatory Leukotriene B_4_ (LTB_4_) (Zhang and Hardwick, 2000). Cytotoxic 1-deoxysphingolipids are metabolized by a cytochrome P450-dependent pathway (Alecu et al., 2017). The cytochrome P450-dependent metabolism of α-tocopherol (vitamin E) was suppressed in NAFLD (Bartolini et al., 2017). *CYP4F2a* mRNA levels significantly increase in steatosis (Figure 6A) and decrease in steatohepatitis, cirrhosis, and HCC. Similarly, P4504F2a protein levels mirror the mRNA results (Figures 6B, C), suggesting that the CYP4F2a P450 is not a significant contributor to 20-HETE levels in the progression of MASLD (Figures 3A, B).

### 3.4 GPR75

**FIGURE 7:**
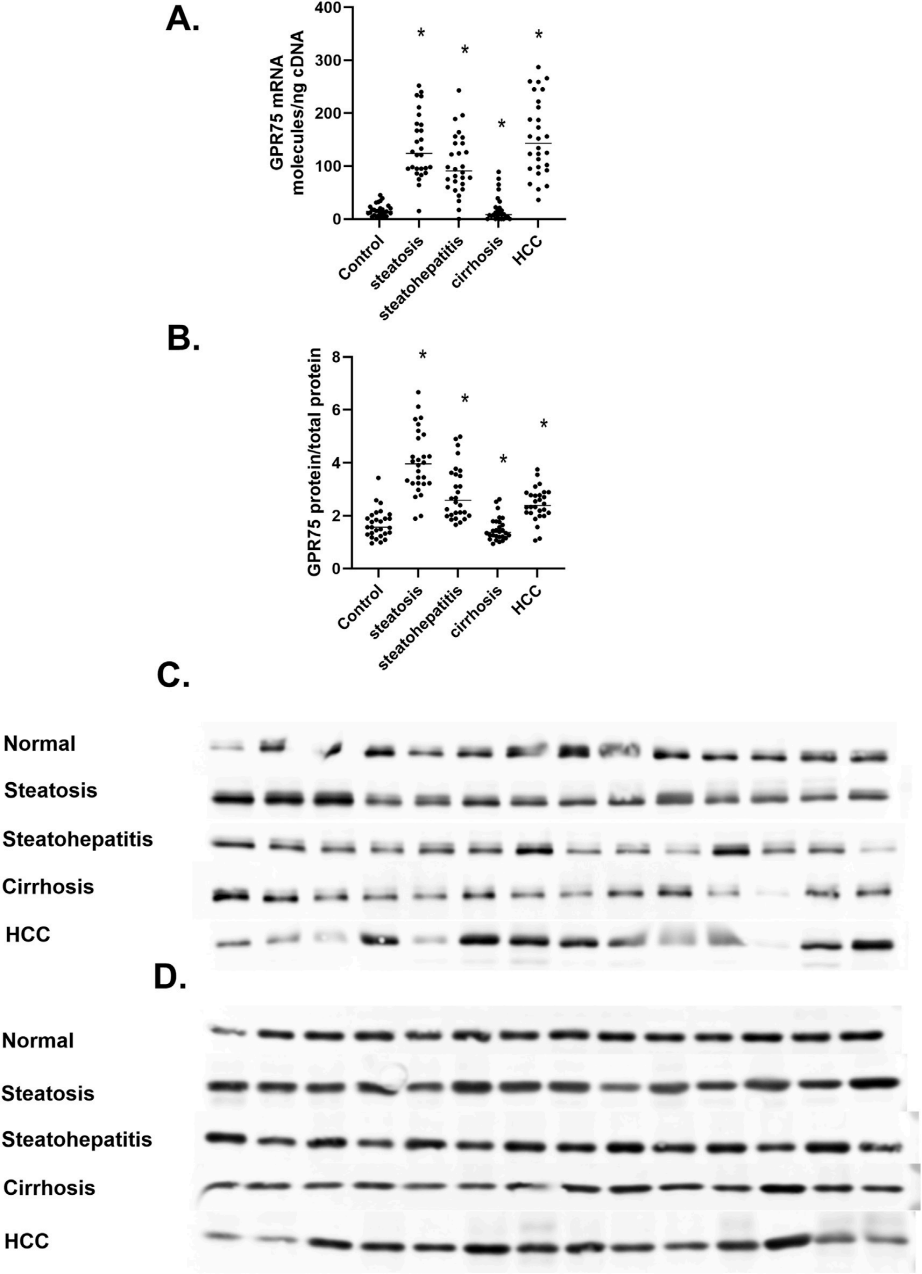
The Regulation of GPR75 mRNA and protein in MASLD. **(A)** *GRP75* mRNA expressed as mRNA molecules per nanogram cDNA, **(B)** The Protein levels of GPR75, **(C)** Western immunoblot analysis of GPR75 protein at different stages of MASLD, **(D)** Level of β-actin protein at different stages of MASLD n = 28 per group, 14 representative immunoblot samples per group. Statistical significance compared to normal liver *p < 0.05.

GPR75 is recognized as a high-affinity primary receptor for 20-HETE and a low-affinity receptor for CCL5/RANTES that triggers signaling pathways of PI3K/AKT and RAS/MAPK (Garcia et al., 2017; Froogh et al., 2022; Garcia et al., 2016). The 20-HETE GPR75 axis has been implicated in cardiovascular, metabolic syndrome, and cancer through its vasoconstriction, insulin resistance, and proliferative properties (Pascale et al., 2023; Dashti et al., 2023; Murtaza et al., 2022). To determine if the differential regulation of *CYP4A11* 20-HETE in MASLD also includes the 20-HETE target protein or receptor, we measured the level of GPR75 mRNA and protein (Figure 7). We observed a significant upregulation of *GPR75* mRNA in steatosis and steatohepatitis and a substantial downregulation of mRNA in liver cirrhosis (Figure 7A). In contrast, *GPR75* mRNA dramatically increased in hepatocellular carcinoma samples. It is of interest that expression of the *GPR75* gene in the normal liver is 1–10 mRNA molecules/ng cDNA, which agrees with 1–14 *GPR75* TPM in RNAseq databases GSE13561, GSE180882, and GSE114564. Interestingly, GPR75 protein levels mirrored mRNA changes with a 2-fold increase in steatosis but decreased from steatohepatitis to cirrhosis and returned to levels significantly above normal values in HCC (Figures 7B, C), where β-actin was used as a loading control (Figure 7D).

## 4 Discussion

Arachidonic acid (AA) can be metabolized into pro-inflammatory (20-HETE) by CYP4 ω-hydroxylases and anti-inflammatory epoxyeicosatrienoic acids (EETs) by CYP2 epoxygenases (Oktem et al., 2023). Very little information is available about the functional role of 20- HETE in liver pathology. However, 20-HETE regulates liver metabolic activity and hemodynamics (Sacerdoti et al., 1997; Sacerdoti et al., 2015; Sacerdoti et al., 2003). In fact, 20-HETE is a potent activator of PPARα (Kd 0.87 µM), PPARγ (1.7 µM), and GPR75 (Kd 3.75 nM). Therefore, it is thus expected to exert essential functions in lipid homeostasis and control fat-dependent energy supply and metabolism. In addition, it is an important inflammatory mediator and may have an indispensable role in regulating inflammatory diseases (Antoun et al., 2008; Hardwick, 2015). The excretory rates of 20- HETE were several-fold higher than those of PGs and TxB2 in liver cirrhosis patients (Sacerdoti et al., 2015; Sacerdoti et al., 2003). Moreover, 20-HETE-producing enzymes are upregulated in human cancers and may be key changes observed across HCC samples (Alexanian et al., 2012). To understand the role of the CYP4/20- HETE/GPR75 axis in the progression of MASLD, we analyzed the production of 20-HETE in the liver of patients with steatosis, steatohepatitis, cirrhosis, or fatty liver-induced hepatocellular carcinoma. We also quantified the expression of the major 20-HETE-producing CYP4F2 and CYP4A11 during the progression of MASLD. Furthermore, we analyzed the expression levels of GPR75, the central receptor for 20-HETE, in patients with MASLD.

We extensively characterized our cohort of liver samples both histologically and metabolically (Figure 2). Throughout this characterization, we observed dynamic stages of MASLD with distinct characteristic changes in metabolic factors/values concerning FFAs and elevated triglycerides in patients with steatosis (Figure 2A). At the same time, the decreased levels of cholesterol and bilirubin (Figures 2B, C) may reflect increased serum levels of VLDL and bilirubin. Serum bilirubin is used as a marker of MELD with three objective variables (i.e., serum bilirubin, serum creatinine, and prothrombin international normalized ratio- INR) (Wiesner et al., 2003). We also observed an increase in bile acids during the progression of MASLD, most likely due to increased activity of cholesterol metabolizing CYP7A1 and CYP27A1 (Figure 2D). The measurement of bile acids may be a critical, underappreciated marker of CLD (Cruz-Ramon et al., 2017). During inflammation of the liver in steatohepatitis, myeloperoxidase activity increases, indicating immune cell infiltration activation and/or infiltration (Figure 2G), and ROS and lipid peroxidation increase in the progression of MASLD (Figures 2E, F). Finally, we found increased hydroxyproline levels as a fibrosis marker in the steatohepatitis, cirrhosis, and HCC groups (Figure 2H). These biochemical data confirm the stages of MASLD progression in our male and female liver samples.

A prominent increase in liver levels of 20-HETE was observed in steatosis to liver cirrhosis. In contrast, its level slightly dropped in HCC (Figure 3A). To determine if the liver level of 20-HETE is due to the ω-hydroxylation of AA by cytochrome P450, we measured the rate of 20-HETE formation in microsomes (Figure 3B). Compared to normal liver, there is a significant increase in 20-HETE synthesis rates at all stages of MASLD, suggesting that activated P4504A11 and P4504F2, the major ω-hydroxylases of AA are responsible for increased 20-HETE synthesis. The 20-HETE eicosanoid has different functions in diabetes, metabolic syndrome, cirrhosis, and HCC, but its role in the progression of MASLD has not been well established. In diabetes, 20-HETE induces insulin secretion and protects pancreatic islet cells from apoptosis by activating the free fatty acid receptor 1 (FFAR1/GPR40) (Tunaru et al., 2018). In addition, 20-HETE exerts anti-hypertensive effects in the kidney by inhibiting sodium reabsorption (Zhang et al., 2018), but promotes vasoconstriction of smooth muscle cells (Fang et al., 2007). Vascular endothelial progenitor cells express high levels of CYP4A11 and 20-HETE that promote angiogenesis (Chen et al., 2014). Elevated 20-HETE levels are observed in patients with liver cirrhosis (Sacerdoti et al., 1997), and 20-HETE is highly expressed in patients with hepatic fibrosis (Li et al., 2023). In proliferating cancer cells, 20-HETE levels increase the level of growth factors such as vascular endothelial growth factor (VEGF), epidermal growth factor (EGF), fibroblast growth factor (FGF), and platelet-derived growth factor (PDGF).

*CYP4A/4F* genes and 20-HETE have been implicated in several cancers (Panigrahy et al., 2010), including ovarian (Alexanian et al., 2012), prostate (Leeson-Payne et al., 2024; Cardenas et al., 2020), lung cancer (Oktem et al., 2023), glioma brain cancer (Guo et al., 2005), and hepatocellular carcinoma (Guo et al., 2005; Eun et al., 2019; Eun et al., 2018). Our data indicate that both *CYP4A11*, *CYP4A22*, and *CYP4F2* mRNA levels increase in steatosis (Figures 4A, B, 5A) while both *CYP4A11* and *CYP4A22* increase in steatohepatitis. However, there is a statistically significant decrease in *CYP4F2* mRNA and protein (Figures 6A, B). In liver samples of cirrhosis, *CYP4A22* increases while *CYP4A11* and *CYP4F2* decrease (Figures 4A, B). In HCC, both *CYP4F2* mRNA and protein levels decrease, while *CYP4A22* and *CYP4A11* mRNA and protein levels increase significantly. These data indicate a differential regulation of the *CYP4* gene isoforms in the progression of MASLD.

Alongside 20-HETE changes to its high-affinity receptor, GPR75 at the G*PR75* mRNA and protein level were measured in the progression of MASLD (Figure 7). GPR75 mRNA and protein increased in steatosis and steatohepatitis, then dropped significantly in cirrhosis but rebounded in HCC samples. The parallels in regulating *CYP4A11* and GPR75 suggest a regulatory link between these genes. The drop in both mRNA and protein in hepatic cirrhosis may be due to desensitization of the GPR75 receptor by increased levels of 20-HETE or the conversion of 20-HETE to 20-carboxyl- arachidonic acid (20-COOH-AA) that functions as an activator of PPARα and PPARγ (Fang et al., 2007) that may suppress *CYP4A11* and GPR75 gene regulation. It is also possible that in cirrhosis, the level of CCL5/RNATES increases to antagonize 20-HETE, leading to a decrease in GPR75 synthesis (Pascale et al., 2021). It will be interesting to test these hypotheses and evaluate the molecular underpinnings involved in the coordinated regulation of the CYP4A11 and GPR75 genes in cell culture and animal models.

To date, this is the first report to determine the level and synthesis of 20-HETE in conjunction with changes in the expression of the major AA ω-hydroxylase *CYP4A/4F* and changes in the expression of the 20-HETE receptor GPR75. We hypothesize that the CYP4A11/20-HETE-dependent activation of the GPR75 receptor drives fundamental mechanisms across the dynamic stages of MASLD-dependent, altering unique inflammatory and metabolic pathways. The importance of the CYP4/20-HETE/GPR75 axis in the liver has yet to be fully uncovered. At the same time, the various human GPR75 variants are protected from obesity, metabolic syndrome, and steatosis (Fragner et al., 2024). It is unclear whether these variants can sustain a level of protection from chronic liver disease and hepatocellular carcinoma. Given 20-HETE vasoconstrictive and pro-inflammatory bioactions, 20-HETE and GPR75 may play a significant role in the pathogenesis of portal hypertension observed during cirrhosis, portal vein thromboses (PVT), and splanchnic thromboses. The dynamic and spatial changes observed across these clinical samples highlight the need to better understand each stage of liver disease. This insight will allow the effective implementation of therapeutic interventions that target the 20-HETE/GPR75 axis, including pharmacological receptor blockers, 20-HETE synthesis inhibitors, and gene-silencing approaches.

## Data Availability

The data underlying this study’s findings are accessible through the corresponding author upon reasonable request. Certain datasets may not be shared to safeguard privacy or comply with ethical restrictions.

## Supplementary material

SUPPLEMENTARY TABLE S1 Supplementary information PCR Primer sets.

SUPPLEMENTARY TABLE S2 Peptide-specific antibodies.

## Glossary

AA: arachidonic acid
AC: Eangiotensin-converting enzymes
ACLD: acute on chronic liver disease
BMI: body mass index
BSEP: bile salt export pump
CCL5: C-C motif chemokine ligand 5
CLD: chronic liver disease
CYP4: cytochrome P450 family 4
11,12 DiHETrE: 11,12-dihydroxy-5Z,8Z,14Z-eicosatrienoic acid
ECM: extracellular matrix
EET: epoxyeicosatrienoic acid
EGF: epidermal growth factor
FGF: fibroblast growth factor
GPR75: G-coupled protein receptor 75
FFAR1: free fatty acid receptor 1
GSE: gene-expression data sets
HCC: hepatocellular carcinoma
HSC: hepatic stellate cells
20- HETE: 20-hydroxyeicosatetraenoic acid
12-HHT: 12-(S)-hydroxy-5Z,8E,10E-heptadecatriaenoic acid
HODE: 13(S)-hydroxy-9Z,11E-octadecadienoic acid
LRE: Linear regression of efficiency
LSEC: liver sinusoidal endothelial cells
LTE4: Leukotriene E4
MAPK: mitogen-activated protein kinase
MASLD: metabolic dysfunction-associated steatotic liver disease
MCD: methionine choline-deficient diet
MRP2: multi-drug resistance associated transport protein 2
NAFLD: non-alcoholic fatty liver disease
NASH: non-alcoholic steatohepatitis
NEB: New England Biolabs
NEFA: non-esterified fatty acids
NSBB: non-selective beta blockers
PPARα: peroxisome proliferator-activated receptor alpha
PPARγ: Peroxisome proliferator-activated receptor gamma
PI3K/Akt: PI3K activation phosphorylates and activates AKT (protein kinase B)
PVT: portal vein thrombosis
RANTES (CCL5): chemokine C-C motif ligand 5 regulated on activation of normal T cell expressed and secreted
RAS: renin-angiotensin system
VEGF: vascular endothelial growth factor

## References

[B1] Akbari P. Gilani A. Sosina O. Kosmicki J. A. Khrimian L. Fang Y. Y. (2021). Sequencing of 640,000 exomes identifies *GPR75* variants associated with protection from obesity. Science 373, eabf8683. 10.1126/science.abf8683 34210852 PMC10275396

[B2] Alecu I. Othman A. Penno A. Saied E. M. Arenz C. von Eckardstein A. (2017). Cytotoxic 1-deoxysphingolipids are metabolized by a cytochrome P450-dependent pathway. J. Lipid Res. 58, 60–71. 10.1194/jlr.M072421 27872144 PMC5234722

[B3] Alexanian A. Miller B. Roman R. J. Sorokin A. (2012). 20-HETE-producing enzymes are up-regulated in human cancers. Cancer Genomics Proteomics 9, 163–169. 22798501 PMC3601443

[B4] Alshehade S. A. (2024). Resmetirom's approval: highlighting the need for comprehensive approaches in NASH therapeutics. Clin. Res. Hepatol. Gastroenterol. 48, 102377. 10.1016/j.clinre.2024.102377 38772519

[B5] Antoun J. Goulitquer S. Amet Y. Dreano Y. Salaun J. P. Corcos L. (2008). CYP4F3B is induced by PGA1 in human liver cells: a regulation of the 20-HETE synthesis. J. Lipid Res. 49, 2135–2141. 10.1194/jlr.M800043-JLR200 18566475

[B6] Baker S. C. Bauer S. R. Beyer R. P. Brenton J. D. Bromley B. Burrill J. (2005). The external RNA controls consortium: a progress report. Nat. Methods 2, 731–734. 10.1038/nmeth1005-731 16179916

[B7] Bartolini D. Torquato P. Barola C. Russo A. Rychlicki C. Giusepponi D. (2017). Nonalcoholic fatty liver disease impairs the cytochrome P-450-dependent metabolism of α-tocopherol (vitamin E). J. Nutr. Biochem. 47, 120–131. 10.1016/j.jnutbio.2017.06.003 28628909

[B8] Cardenas S. Colombero C. Cruz M. Mormandi E. Adebesin A. M. Falck J. R. (2023). 20-HETE/GPR75 pairing modulates the expression and transcriptional activity of the androgen receptor in androgen-sensitive prostate cancer cells. Mol. Cell Endocrinol. 559, 111784. 10.1016/j.mce.2022.111784 36202260

[B9] Cardenas S. Colombero C. Panelo L. Dakarapu R. Falck J. R. Costas M. A. (2020). GPR75 receptor mediates 20-HETE-signaling and metastatic features of androgen-insensitive prostate cancer cells. Biochim. Biophys. Acta Mol. Cell Biol. Lipids 1865, 158573. 10.1016/j.bbalip.2019.158573 31760076 PMC6957769

[B10] Chen L. Ackerman R. Saleh M. Gotlinger K. H. Kessler M. Mendelowitz L. G. (2014). 20-HETE regulates the angiogenic functions of human endothelial progenitor cells and contributes to angiogenesis *in vivo* . J. Pharmacol. Exp. Ther. 348, 442–451. 10.1124/jpet.113.210120 24403517 PMC3935142

[B11] Chopra A. Willmore W. G. Biggar K. K. (2019). Protein quantification and visualization via ultraviolet-dependent labeling with 2,2,2-trichloroethanol ultraviolet-dependent labeling with 2,2,2-trichloroethane. Sci. Rep. 9 13923. 10.1038/s41598-019-50385-9 31558752 PMC6763483

[B12] Cruz-Ramon V. Chinchilla-Lopez P. Ramirez-Perez O. Mendez-Sanchez N. (2017). Bile acids in non-alcoholic fatty liver disease: new concepts and therapeutic advances. Ann. Hepatol. 16, s58–s67. 10.5604/01.3001.0010.5498 29080343

[B13] Dashti M. R. Gorbanzadeh F. Jafari-Gharabaghlou D. Farhoudi Sefidan Jadid M. Zarghami N. (2023). G protein-coupled receptor 75 (GPR75) as a novel molecule for targeted therapy of cancer and metabolic syndrome. Asian Pac J. Cancer Prev. 24, 1817–1825. 10.31557/APJCP.2023.24.5.1817 37247305 PMC10495892

[B14] Devarbhavi H. Asrani S. K. Arab J. P. Nartey Y. A. Pose E. Kamath P. S. (2023). Global burden of liver disease: 2023 update. J. Hepatol. 79, 516–537. 10.1016/j.jhep.2023.03.017 36990226

[B15] Duan X. Zhang Y. Xu T. (2024). CYP4A22 loss-of-function causes a new type of vitamin D-dependent rickets (VDDR1C). J. Bone Min. Res. 39, 967–979. 10.1093/jbmr/zjae084 38847469

[B16] Durairaj P. Fan L. Machalz D. Wolber G. Bureik M. (2019). Functional characterization and mechanistic modeling of the human cytochrome P450 enzyme CYP4A22. F.E.B.S. Lett. 593, 2214–2225. 10.1002/1873-3468.13489 31199497

[B17] Eun H. S. Cho S. Y. Lee B. S. Kim S. Song I. S. Chun K. (2019). Cytochrome P450 4A11 expression in tumor cells: a favorable prognostic factor for hepatocellular carcinoma patients. J. Gastroenterol. Hepatol. 34, 224–233. 10.1111/jgh.14406 30069903

[B18] Eun H. S. Cho S. Y. Lee B. S. Seong I. O. Kim K. H. (2018). Profiling cytochrome P450 family 4 gene expression in human hepatocellular carcinoma. Mol. Med. Rep. 18, 4865–4876. 10.3892/mmr.2018.9526 30280198 PMC6236316

[B19] Fang X. Dillon J. S. Hu S. Harmon S. D. Yao J. Anjaiah S. (2007). 20- carboxy-arachidonic acid is a dual activator of peroxisome proliferator-activated receptors alpha and gamma. Prostagl. Other Lipid Mediat 82, 175–184. 10.1016/j.prostaglandins.2006.05.002 17164145

[B20] Feldstein A. E. Lopez R. Tamimi T. A. Yerian L. Chung Y. M. Berk M. (2010). Mass spectrometric profiling of oxidized lipid products in human nonalcoholic fatty liver disease and nonalcoholic steatohepatitis. J. Lipid Res. 51, 3046–3054. 10.1194/jlr.M007096 20631297 PMC2936759

[B21] Fragner M. L. Parikh M. A. Jackson K. A. Schwartzman M. L. Frishman W. H. Peterson S. J. (2024). GPR75: a newly identified receptor for targeted intervention in the treatment of obesity and metabolic syndrome. Cardiol. Rev. 10.1097/CRD.0000000000000711 PMC11808825 38695569

[B22] Froogh G. Garcia V. Laniado Schwartzman M. (2022). The CYP/20-HETE/GPR75 axis in hypertension. Adv. Pharmacol. 94, 1–25. 10.1016/bs.apha.2022.02.003 35659370 PMC10123763

[B23] Garcia V. Gilani A. Shkolnik B. Pandey V. Zhang F. F. Dakarapu R. (2017). 20- HETE signals through G-protein-coupled receptor GPR75 (G(q)) to affect vascular function and trigger hypertension. Circ. Res. 120, 1776–1788. 10.1161/CIRCRESAHA.116.310525 28325781 PMC5446268

[B24] Garcia V. Shkolnik B. Milhau L. Falck J. R. Schwartzman M. L. (2016). 20-HETE activates the transcription of angiotensin-converting enzyme via nuclear factor-κb translocation and promoter binding. J. Pharmacol. Exp. Ther. 356, 525–533. 10.1124/jpet.115.229377 26699146 PMC4767392

[B25] Guerra Ruiz A. R. Crespo J. Lopez Martinez R. M. Iruzubieta P. Casals Mercadal G. Lalana Garces M. (2021). Measurement and clinical usefulness of bilirubin in liver disease. Adv. Lab. Med. 2, 352–372. 10.1515/almed-2021-0047 37362415 PMC10197288

[B26] Gunarathne L. S. Rajapaksha H. Shackel N. Angus P. W. Herath C. B. (2020). Cirrhotic portal hypertension: from pathophysiology to novel therapeutics. World J. Gastroenterol. 26, 6111–6140. 10.3748/wjg.v26.i40.6111 33177789 PMC7596642

[B27] Guo M. Roman R. J. Falck J. R. Edwards P. A. Scicli A. G. (2005). Human U251 glioma cell proliferation is suppressed by HET0016 [N-hydroxy-N'-(4-butyl-2- methylphenyl)formamidine], a selective inhibitor of CYP4A. J. Pharmacol. Exp. Ther. 315, 526–533. 10.1124/jpet.105.088567 16081682

[B28] Hardwick J. P. (2015). Cytochrome P450 function and pharmacological roles in inflammation and cancer. Preface. Adv. Pharmacol. 74. 10.1016/S1054-3589(15)00047-2 26233914

[B29] Hu X. Du S. Yu J. Yang X. Yang C. Zhou D. (2016). Common housekeeping proteins are up-regulated in colorectal adenocarcinoma and hepatocellular carcinoma, making the total protein a better housekeeper. Oncotarget 7, 66679–66688. 10.18632/oncotarget.11439 27556505 PMC5341829

[B30] Huang D. Q. Terrault N. A. Tacke F. Gluud L. L. Arrese M. Bugianesi E. (2023). Global epidemiology of cirrhosis - aetiology, trends and predictions. Nat. Rev. Gastroenterol. Hepatol. 20, 388–398. 10.1038/s41575-023-00759-2 36977794 PMC10043867

[B31] Jakubek P. Kalinowski P. Karkucinska-Wieckowska A. Kaikini A. Simoes I. C. M. Potes Y. (2024). Oxidative stress in metabolic dysfunction-associated steatotic liver disease (MASLD): how does the animal model resemble human disease? Faseb J. 38, e23466. 10.1096/fj.202302447R 38318780

[B32] Karsdal M. A. Daniels S. J. Holm Nielsen S. Bager C. Rasmussen D. G. K. Loomba R. (2020). Collagen biology and non-invasive biomarkers of liver fibrosis. Liver Int. 40, 736–750. 10.1111/liv.14390 31997561

[B33] Korbie D. J. Mattick J. S. (2008). Touchdown PCR for increased specificity and sensitivity in PCR amplification. Nat. Protoc. 3, 1452–1456. 10.1038/nprot.2008.133 18772872

[B34] Leahy C. Osborne N. Shirota L. Rote P. Lee Y. K. Song B. J. (2024). The fatty acid omega hydroxylase genes (CYP4 family) in the progression of metabolic dysfunction-associated steatotic liver disease (MASLD): an RNA sequence database analysis and review. Biochem. Pharmacol. 228, 116241. 10.1016/j.bcp.2024.116241 38697309 PMC11774579

[B35] Leeson-Payne A. Iyinikkel J. Malcolm C. Lam B. Y. H. Sommer N. Dowsett G. K. C. (2024). Loss of GPR75 protects against non-alcoholic fatty liver disease and body fat accumulation. Cell Metab. 36, 1076–1087 e4. 10.1016/j.cmet.2024.03.016 38653246

[B36] Li B. Ma Y. Tan L. Ren H. Wu L. Su Q. (2023). 20-Hydroxytetraenoic acid induces hepatic fibrosis via the TGF-β1/Smad3 signaling pathway. Toxicol. Lett. 373, 1–12. 10.1016/j.toxlet.2022.11.001 36368619

[B37] Liu T. Wang R. X. Han J. Li Z. D. Sheps J. A. Zheng L. J. (2024). Comprehensive bile acid profiling of ABCB4-mutated patients and the prognostic role of taurine-conjugated 3α,6α,7α,12α-tetrahydroxylated bile acid in cholestasis. J. Clin. Transl. Hepatol. 12, 151–161. 10.14218/JCTH.2023.00095 38343606 PMC10851069

[B38] Lopez-Vicario C. Checa A. Urdangarin A. Aguilar F. Alcaraz-Quiles J. Caraceni P. (2020). Targeted lipidomics reveals extensive changes in circulating lipid mediators in patients with acutely decompensated cirrhosis. J. Hepatol. 73, 817–828. 10.1016/j.jhep.2020.03.046 32294533

[B39] Masarone M. Rosato V. Dallio M. Gravina A. G. Aglitti A. Loguercio C. (2018). Role of oxidative stress in pathophysiology of non-alcoholic fatty liver disease. Oxid. Med. Cell Longev. 2018, 9547613. 10.1155/2018/9547613 29991976 PMC6016172

[B40] Murtaza B. Asghar F. Patoli D. (2022). GPR75: an exciting new target in metabolic syndrome and related disorders. Biochimie 195, 19–26. 10.1016/j.biochi.2022.01.005 35045335

[B41] Oktem E. K. Aydin B. Gulfidan G. Arga K. Y. (2023). A transcriptomic and reverse-engineering strategy reveals molecular signatures of arachidonic acid metabolism in 12 cancers. OMICS 27, 127–138. 10.1089/omi.2022.0185 36800175

[B42] Osborne N. Leahy C. Lee Y. K. Rote P. Song B. J. Hardwick J. P. (2022). CYP4V2 fatty acid omega hydroxylase, a druggable target for the treatment of metabolic associated fatty liver disease (MAFLD). Biochem. Pharmacol. 195, 114841. 10.1016/j.bcp.2021.114841 34798124

[B43] Panigrahy D. Kaipainen A. Greene E. R. Huang S. (2010). Cytochrome P450-derived eicosanoids: the neglected pathway in cancer. Cancer Metastasis Rev. 29, 723–735. 10.1007/s10555-010-9264-x 20941528 PMC2962793

[B44] Pascale J. V. Park E. J. Adebesin A. M. Falck J. R. Schwartzman M. L. Garcia V. (2021). Uncovering the signalling, structure and function of the 20-HETE-GPR75 pairing: identifying the chemokine CCL5 as a negative regulator of GPR75. Br. J. Pharmacol. 178, 3813–3828. 10.1111/bph.15525 33974269 PMC10119890

[B45] Pascale J. V. Wolf A. Kadish Y. Diegisser D. Kulaprathazhe M. M. Yemane D. (2023). 20-Hydroxyeicosatetraenoic acid (20-HETE): bioactions, receptors, vascular function, cardiometabolic disease and beyond. Adv. Pharmacol. 97, 229–255. 10.1016/bs.apha.2023.01.002 37236760 PMC10683332

[B46] Peng Y. Wei Q. Liu Y. Wu Z. Zhang H. Wu H. (2021). Prediction and risk factors for prognosis of cirrhotic patients with hepatic encephalopathy. Gastroenterol. Res. Pract. 2021, 5623601–5623614. 10.1155/2021/5623601 34712321 PMC8546404

[B47] Pfisterer N. Schwarz M. Schwarz C. Putre F. Ritt L. Riedl F. (2024). Statins, metformin, and RAS inhibitors did not reduce variceal bleeding risk and mortality in a large, real-life cohort of patients with cirrhosis. PLoS One 19, e0302811. 10.1371/journal.pone.0302811 38870117 PMC11175511

[B48] Riazi K. Azhari H. Charette J. H. Underwood F. E. King J. A. Afshar E. E. (2022). The prevalence and incidence of NAFLD worldwide: a systematic review and meta-analysis. Lancet Gastroenterol. Hepatol. 7, 851–861. 10.1016/s2468-1253(22)00165-0 35798021

[B49] Rutledge R. G. Stewart D. (2010). Assessing the performance capab0069lities of LRE-based assays for absolute quantitative real-time PCR. PLoS One 5, e9731. 10.1371/journal.pone.0009731 20305810 PMC2840021

[B50] Sacerdoti D. Balazy M. Angeli P. Gatta A. McGiff J. C. (1997). Eicosanoid excretion in hepatic cirrhosis. Predominance of 20-HETE. J. Clin. Invest 100, 1264–1270. 10.1172/JCI119640 9276745 PMC508304

[B51] Sacerdoti D. Gatta A. McGiff J. C. (2003). Role of cytochrome P450-dependent arachidonic acid metabolites in liver physiology and pathophysiology. Prostagl. Other Lipid Mediat 72, 51–71. 10.1016/s1098-8823(03)00077-7 14626496

[B52] Sacerdoti D. Pesce P. Di Pascoli M. Brocco S. Cecchetto L. Bolognesi M. (2015). Arachidonic acid metabolites and endothelial dysfunction of portal hypertension. Prostagl. Other Lipid Mediat 120, 80–90. 10.1016/j.prostaglandins.2015.05.008 26072731

[B53] Singal A. K. Kamath P. S. (2013). Model for end-stage liver disease. J. Clin. Exp. Hepatol. 3, 50–60. 10.1016/j.jceh.2012.11.002 25755471 PMC3940492

[B54] Tunaru S. Bonnavion R. Brandenburger I. Preussner J. Thomas D. Scholich K. (2018). 20-HETE promotes glucose-stimulated insulin secretion in an autocrine manner through FFAR1. Nat. Commun. 9, 177. 10.1038/s41467-017-02539-4 29330456 PMC5766607

[B55] Tunctan B. Senol S. P. Temiz-Resitoglu M. Yilmaz D. E. Guden D. S. Bahceli O. (2022). Activation of GPR75 signaling pathway contributes to the effect of a 20-HETE mimetic, 5,14-HEDGE, to prevent hypotensive and tachycardic responses to lipopolysaccharide in a rat model of septic shock. J. Cardiovasc Pharmacol. 80, 276–293. 10.1097/fjc.0000000000001265 35323151

[B56] Wiesner R. Edwards E. Freeman R. Harper A. Kim R. Kamath P. (2003). Model for end-stage liver disease (MELD) and allocation of donor livers. Gastroenterology 124, 91–96. 10.1053/gast.2003.50016 12512033

[B57] Xie W. Cao Y. Xu M. Wang J. Zhou C. Yang X. (2017). Prognostic significance of elevated cholestatic enzymes for fibrosis and hepatocellular carcinoma in hospital discharged chronic viral hepatitis patients. Sci. Rep. 7, 10289. 10.1038/s41598-017-11111-5 28860489 PMC5579038

[B58] Zhang C. Booz G. W. Yu Q. He X. Wang S. Fan F. (2018). Conflicting roles of 20-HETE in hypertension and renal end-organ damage. Eur. J. Pharmacol. 833, 190–200. 10.1016/j.ejphar.2018.06.010 29886242 PMC6057804

[B59] Zhang X. Hardwick J. P. (2000). Regulation of CYP4F2 leukotriene B4 omega-hydroxylase by retinoic acids in HepG2 cells. Biochem. Biophys. Res. Commun. 279, 864–871. 10.1006/bbrc.2000.4020 11162441

[B60] Zhang X. Li S. Zhou Y. Su W. Ruan X. Wang B. (2017). Ablation of cytochrome P450 omega-hydroxylase 4A14 gene attenuates hepatic steatosis and fibrosis. Proc. Natl. Acad. Sci. U. S. A. 114, 3181–3185. 10.1073/pnas.1700172114 28270609 PMC5373383

