# Supplementary figures and images for: The CYP4/20-HETE/GPR75 axis in the progression of metabolic dysfunction-associated steatosis liver disease (MASLD) to chronic liver disease
Source: Front Physiol. 2025 Jan 29;15:1497297. doi: 10.3389/fphys.2024.1497297 (PMC11826315; doi:10.3389/fphys.2024.1497297)

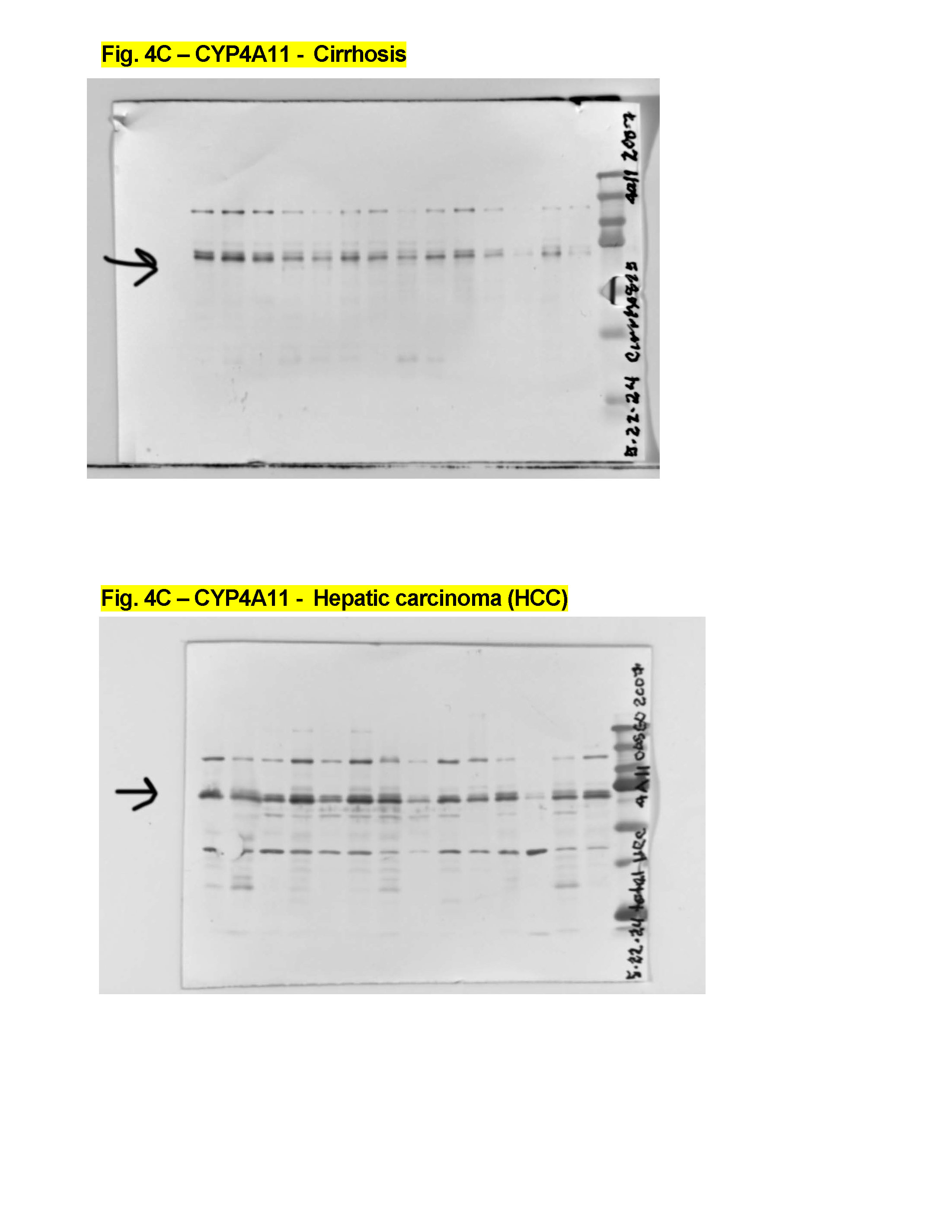

Supplement: Supplementary file 1 [file Image1.tiff]

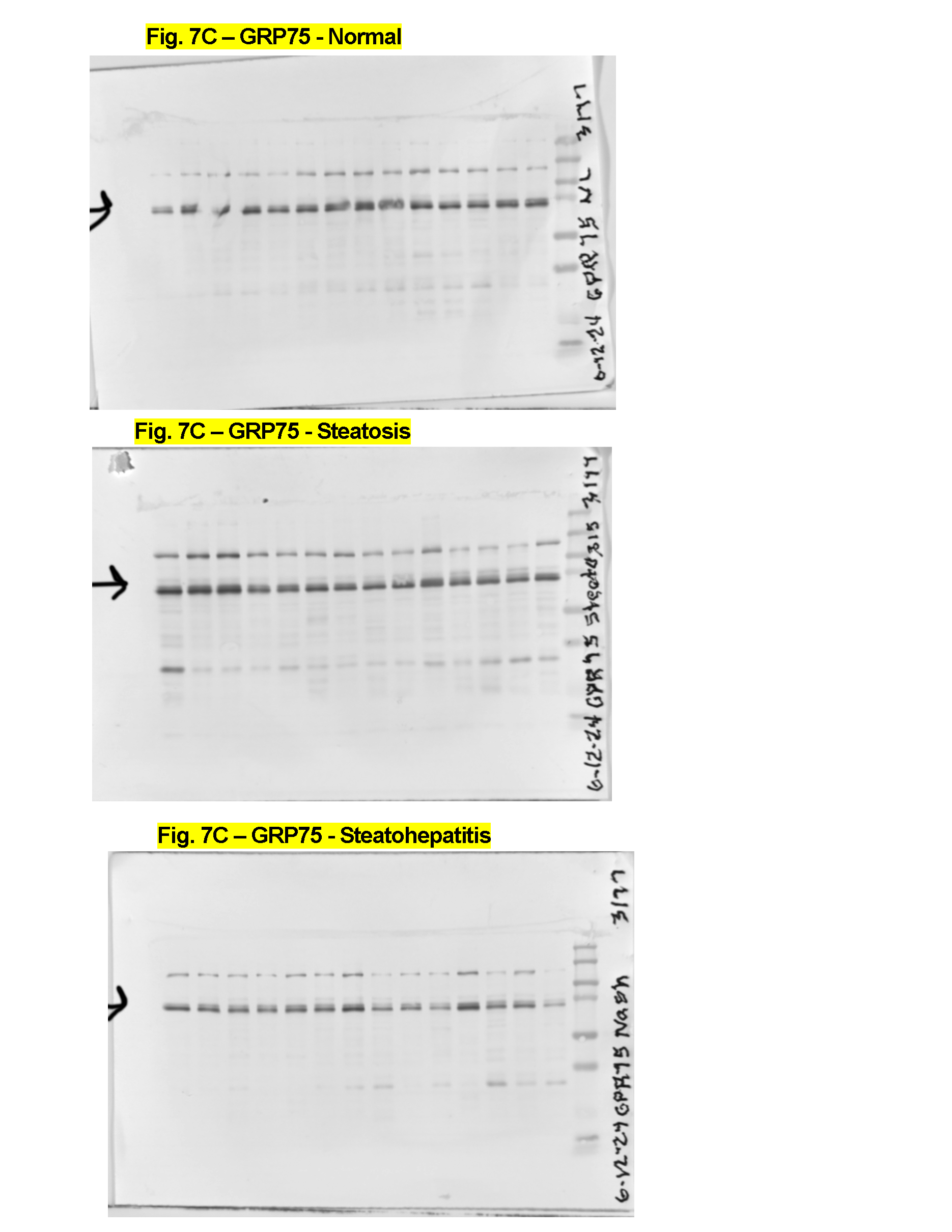

Supplement: Supplementary file 2 [file Image9.tiff]

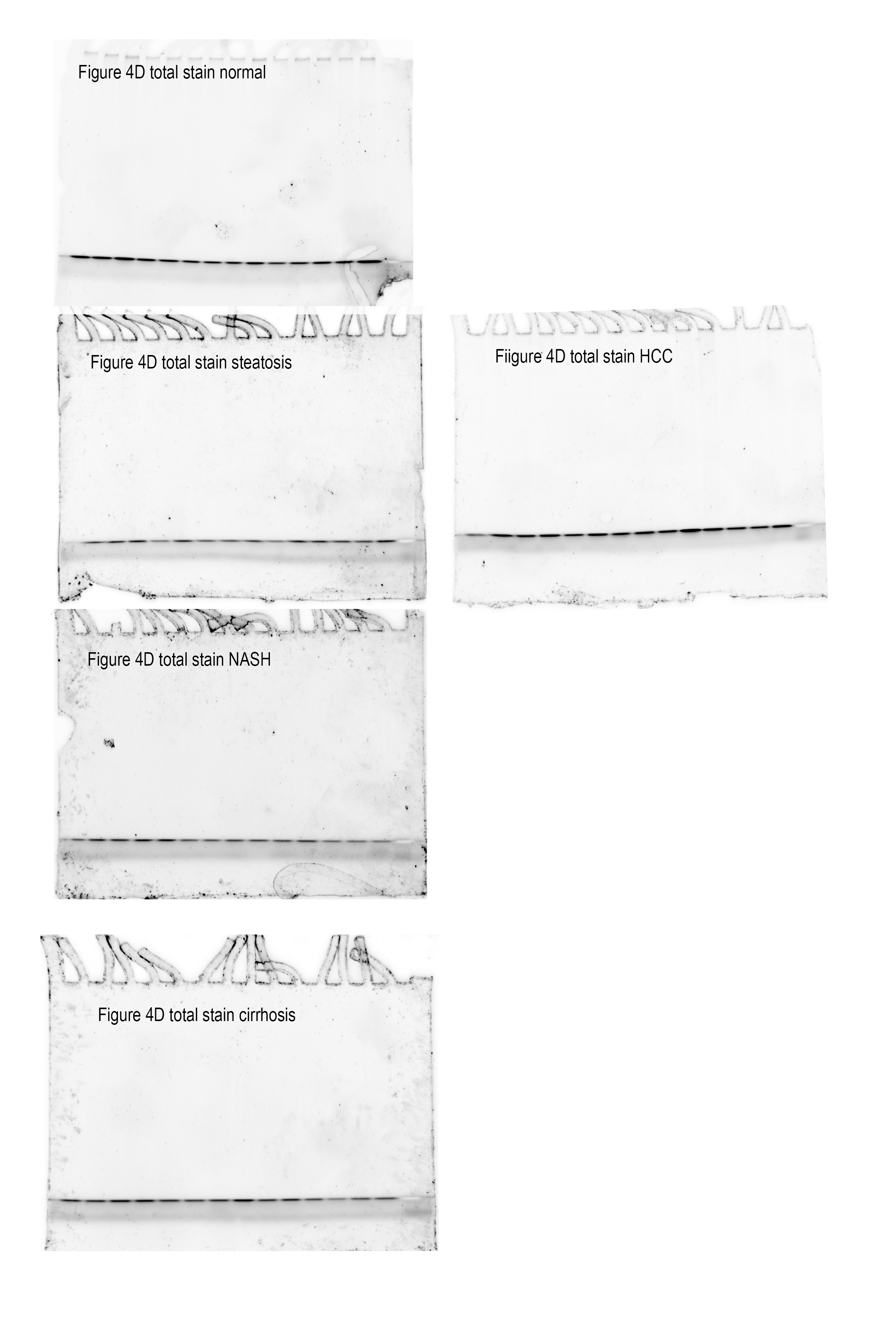

Supplement: Supplementary file 4 [file Image3.tif]

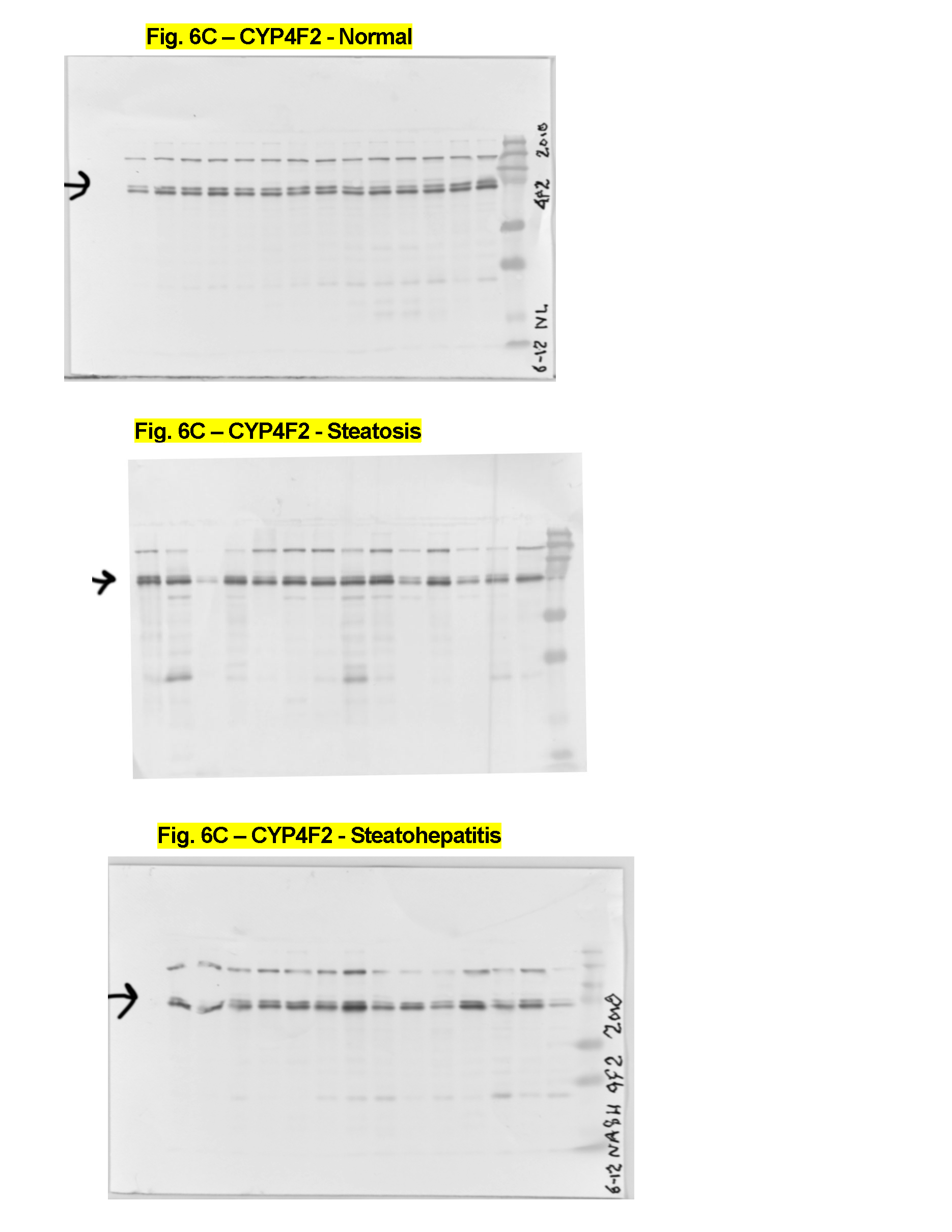

Supplement: Supplementary file 5 [file Image5.tiff]

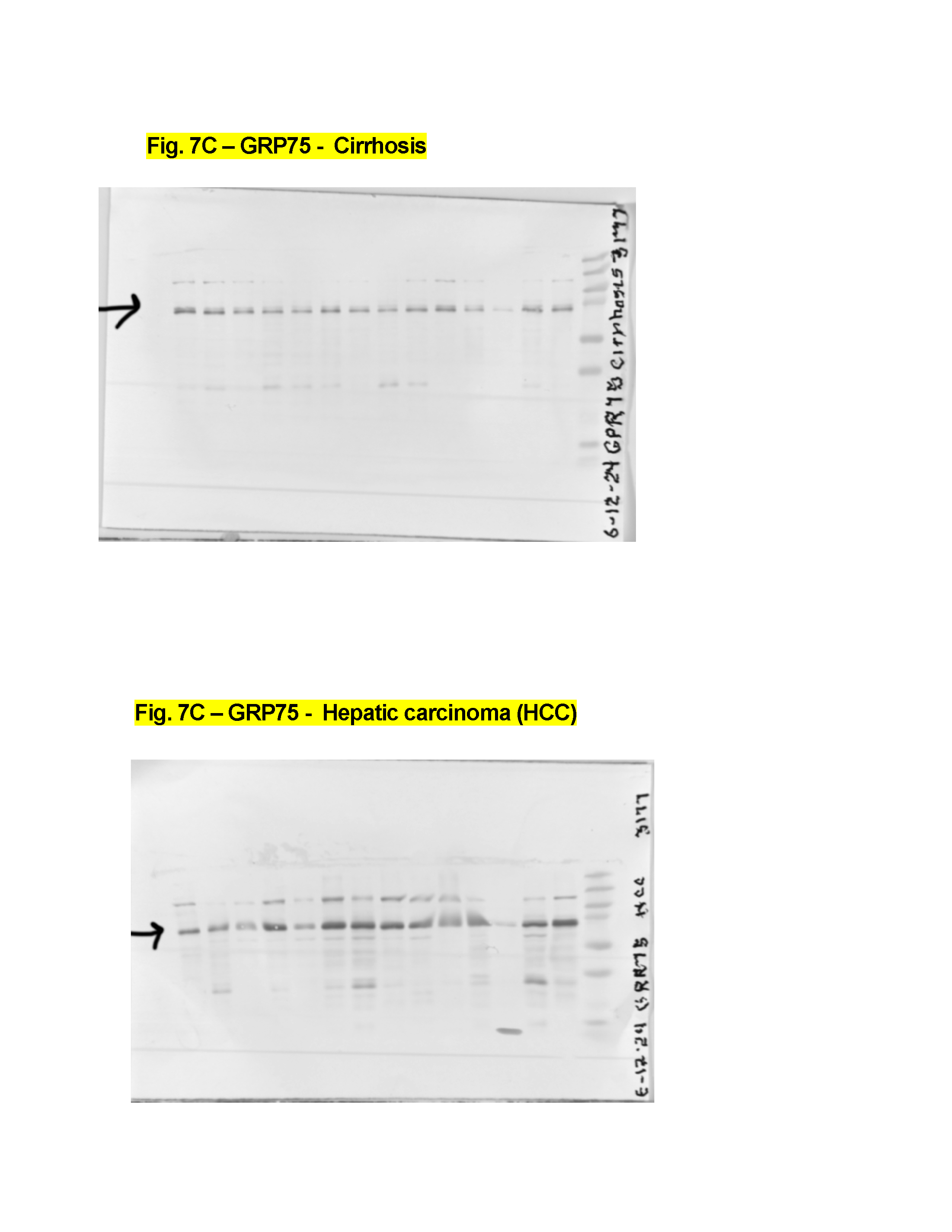

Supplement: Supplementary file 6 [file Image8.tiff]

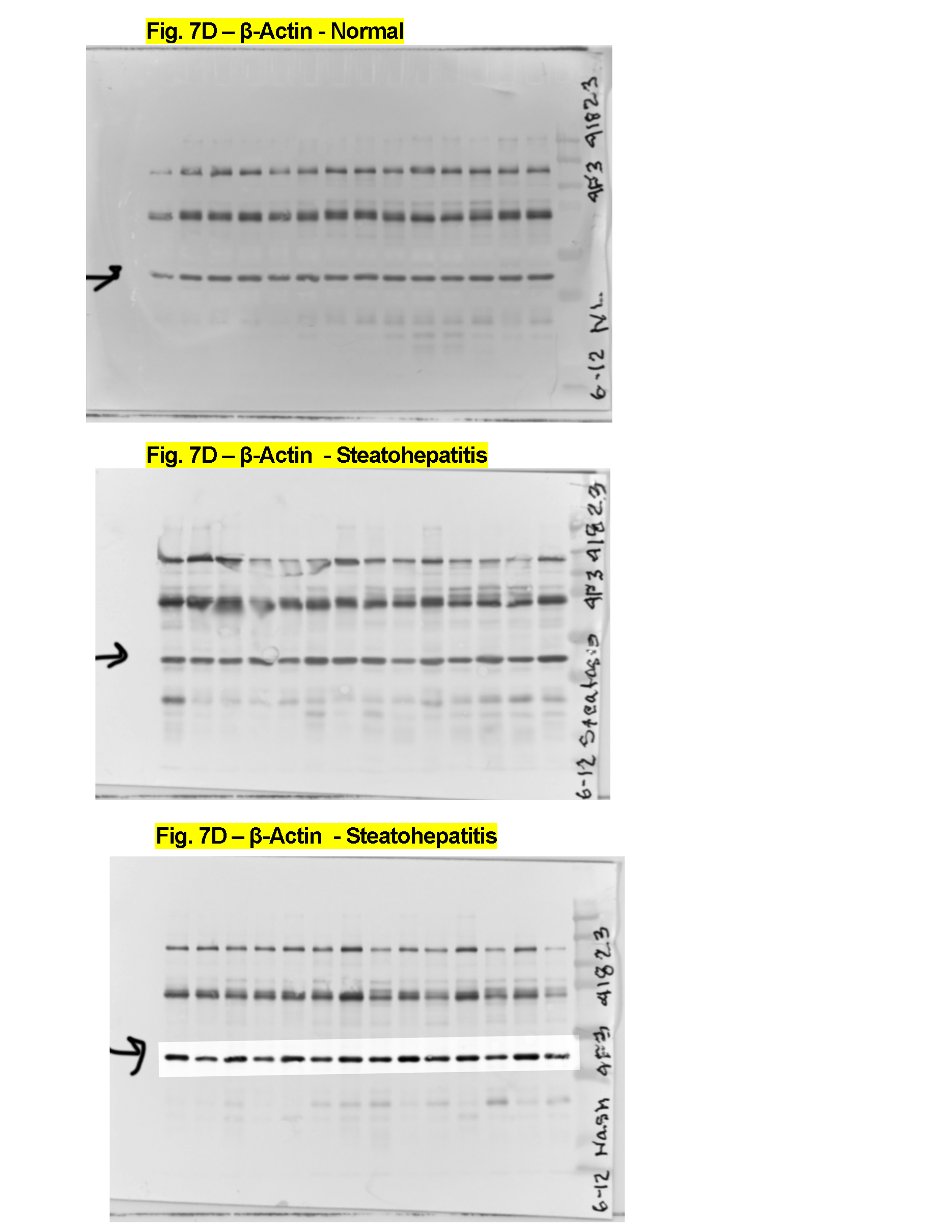

Supplement: Supplementary file 7 [file Image11.tiff]

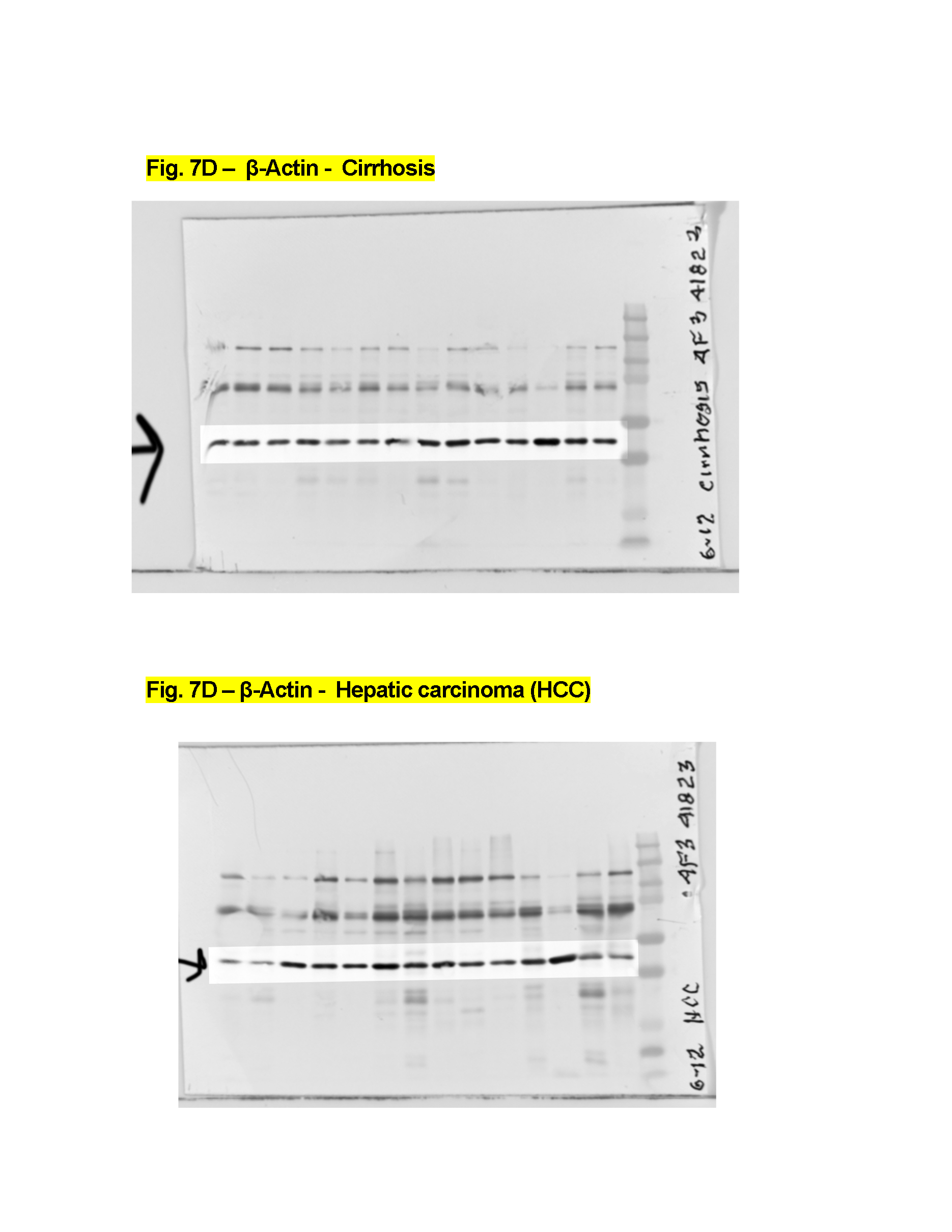

Supplement: Supplementary file 8 [file Image10.tiff]

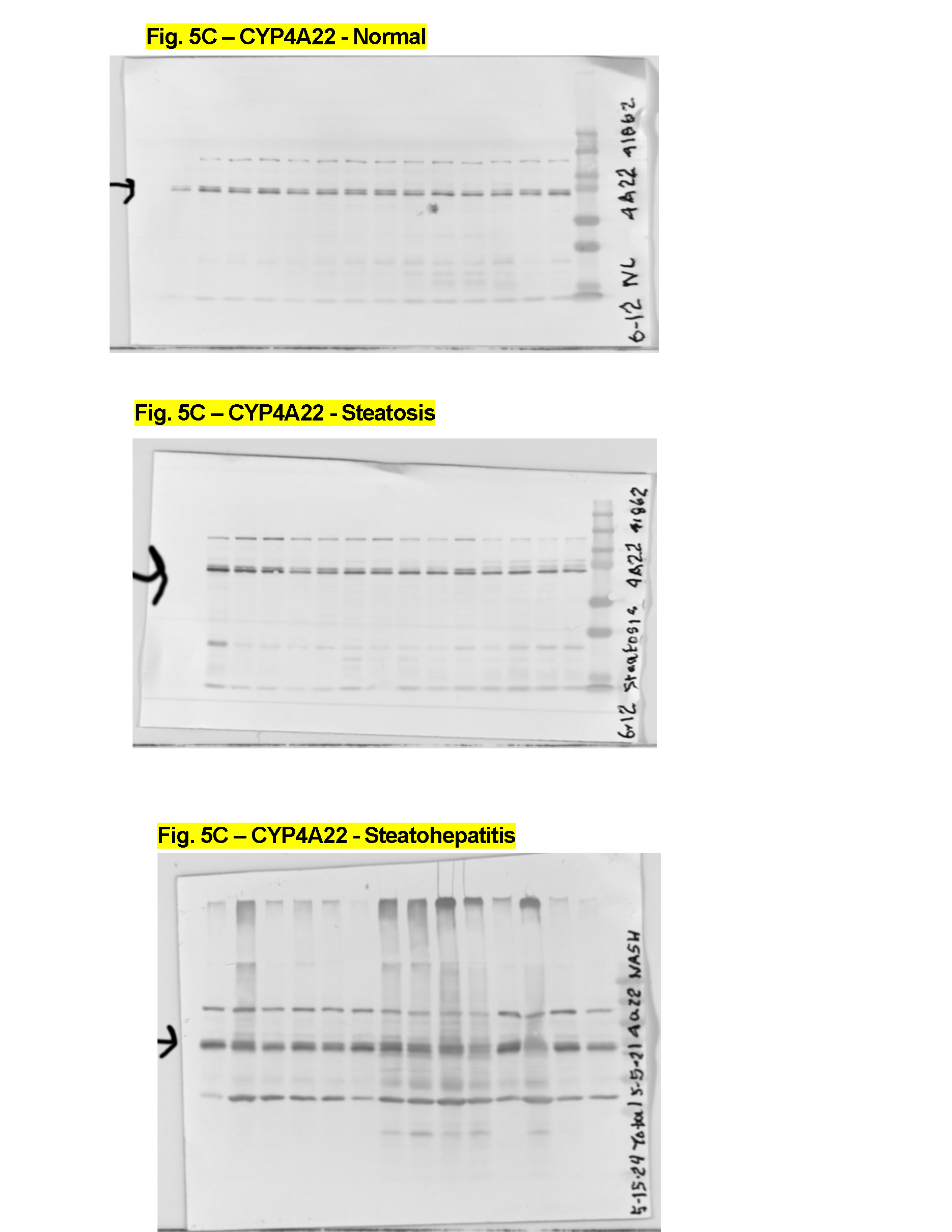

Supplement: Supplementary file 9 [file Image6.tiff]

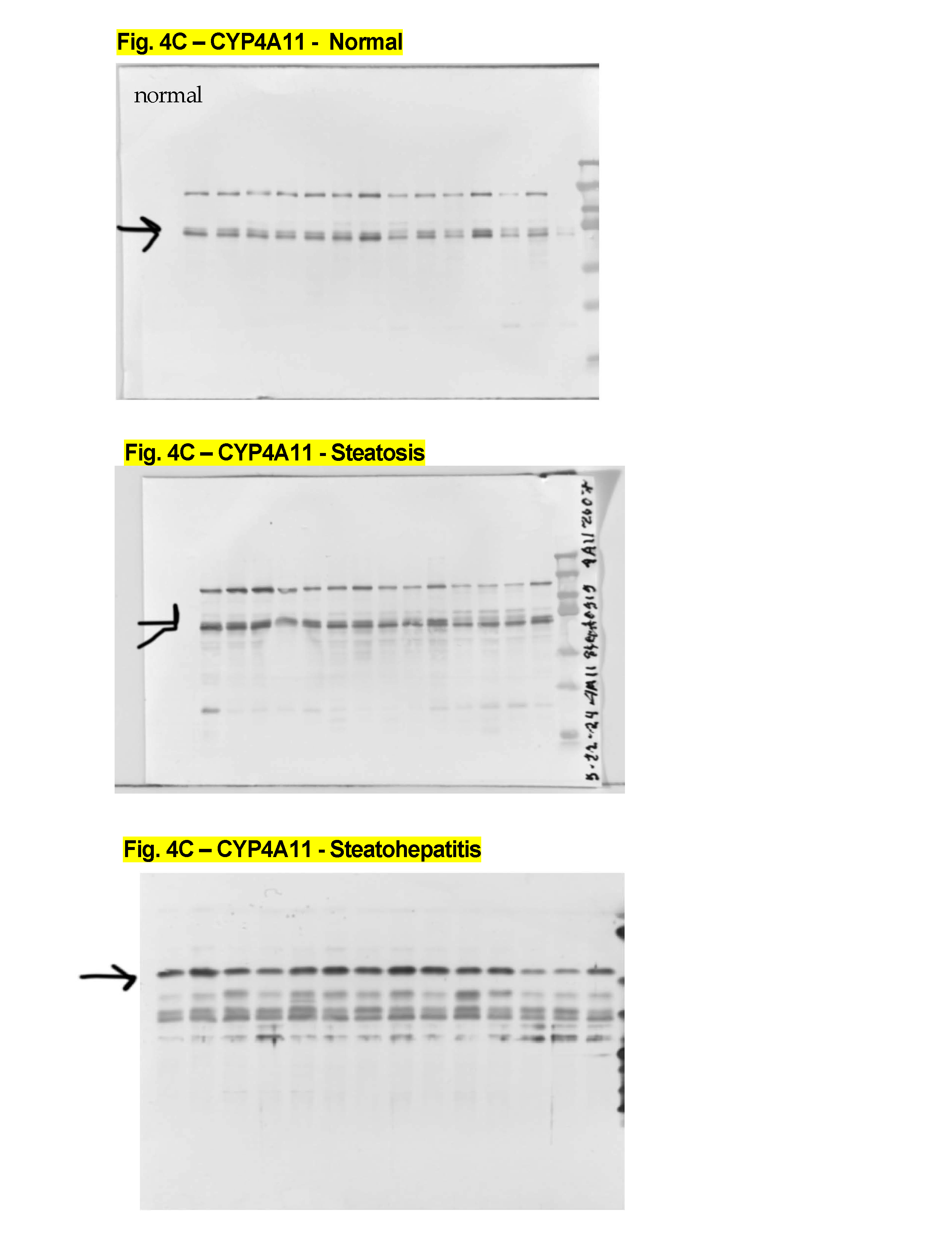

Supplement: Supplementary file 10 [file Image2.tiff]

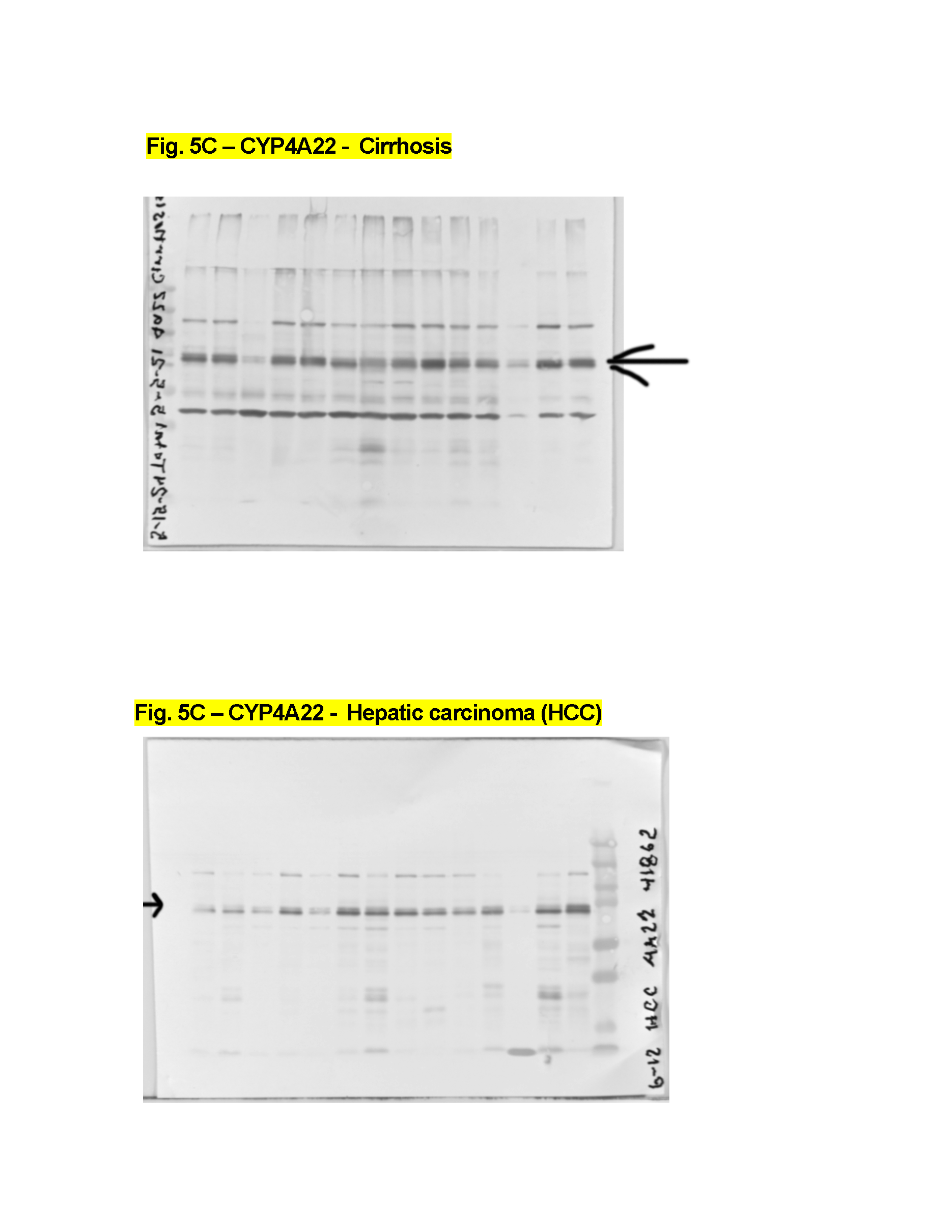

Supplement: Supplementary file 11 [file Image4.tiff]

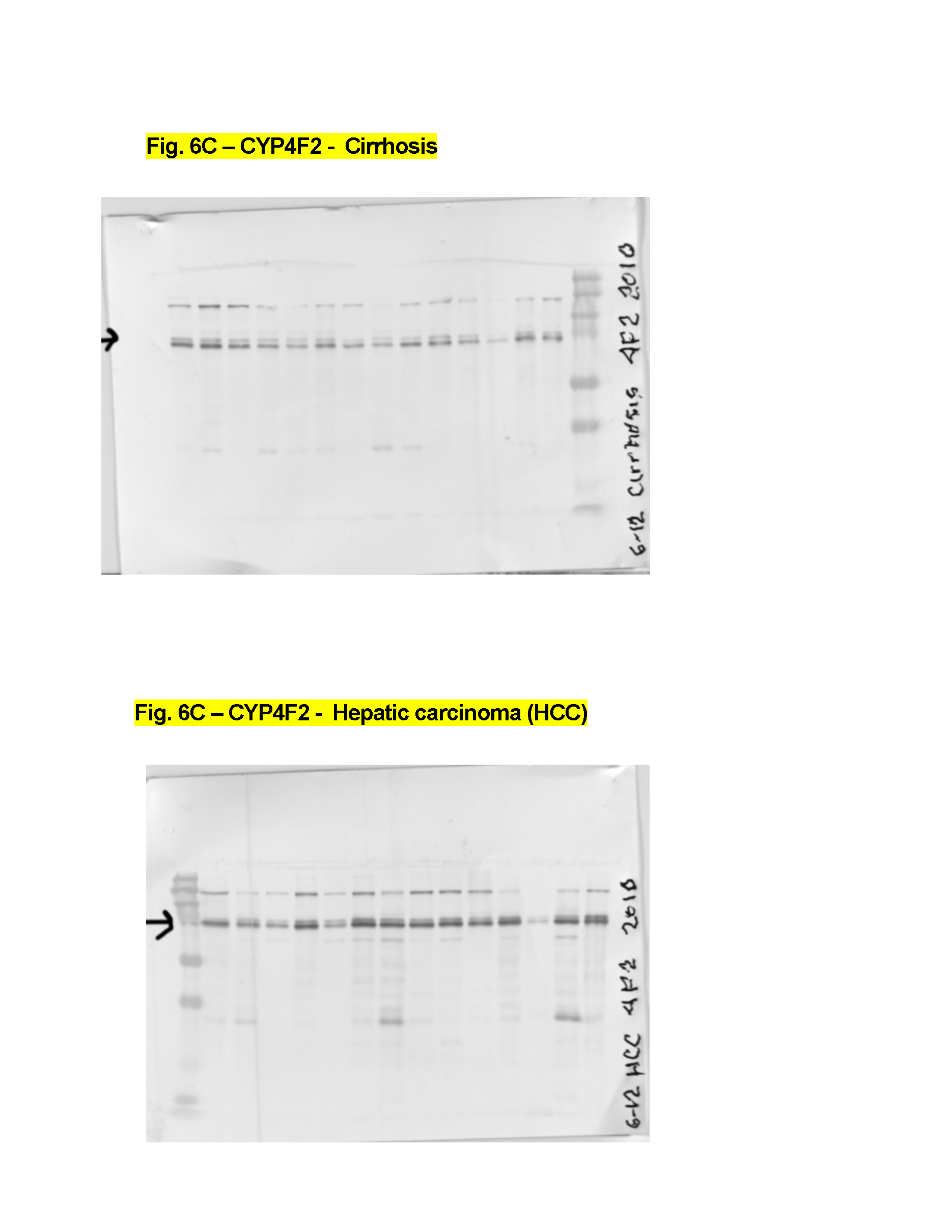

Supplement: Supplementary file 12 [file Image7.tiff]
