# Supplementary material for: The CYP4/20-HETE/GPR75 axis in the progression of metabolic dysfunction-associated steatosis liver disease (MASLD) to chronic liver disease
Source: Front Physiol. 2025 Jan 29;15:1497297. doi: 10.3389/fphys.2024.1497297 (PMC11826315; doi:10.3389/fphys.2024.1497297)
Supplement: Supplementary file 3 [file Table1.docx]

**Supplementary Information**

**Supplementary Table 1: PCR Primer Sets**

**GPR75 NM_00679.4**

**hGRP75 F129** GCC ACC CGG CAG GCT TAT CT

**hGPR75 R248** TTC GGA GAG AAA TGT CTC CTT C

**GPR75 NM_006794**

**hGPR75 F51** TGTGCCTCACTCACAGGAAG

**hGPR75 R135** AGATGACCGCCAGTAGAAAAGTA

**4A11 F717 NM00778** ACAACCTGGTTTTTTCCCGTG

**4A11 R754 NM00778** GGCTGTAGATGGTGTCATTCTGG

**4A11 NM_000778.4 F1558** TCTCAGGAGGCTCCCTAACC

**4a11 NM_000778.4 R1666** TATGGGCAGACAGGAAGGGG

**4A11 NM_000778.4 F1558** TCTCAGGAGGCTCCCTAACC

**4A11 NM_00101969 R1637** GGTCAGGAAGACAGGATGGC

**4A22 NM_001010969 F1554** CCATGGCACGACTTGTGTTG

**4a22 NM_001010969 R1647** TAGGAGTGGGGGTCAGGAAG

**4A22 NM_001010969 F1554** CCATGGCACGACTTGTGTTG

**4A22 NM_00101969 F1560** TCTCAGGAGGCTCCCTAACC

**4F2A NM_001082 F245** GTTTCCCACAACCCCCAAG

**4F2A NM_001082 R292** GGGGTGGCACAAACTGAGGAG

**4F2a NM_001082 F214** CAAGACGGAACTGGTTTTGGGG

**4F2A NM_001082 R283** GTCAGAACTCTCATGCCCTCC

**Supplementary Table 2: Peptides and Specific Antibodies**

**P4504A11 Company: Aviva, catalog: ARP84477**

MKCAFSHQGSIQVDRNSQSYIQAISDLNNLVFSRVRNAFHQNDTIYSLTS

**P4504A11 Company: Aviva, catalog: OAAB04961**

GIMVLLSIYGLHHNPKYWPNPEVFDPFR

**P4504A22 Company: A.V.I.V.A., catalog: ARP41812**

MSVSVLSPSRRLGGVSGILQVTSLLILLLLLIKAAQLYLHRQWLLKALQQ

**P4504A22 Company: A.V.I.V.A., catalog: ARP41862**

AQLYLHRQWLLKALQQFPCPPSHWLFGHIQEFQHDQELQRIQERVKTFPS

**P4504F2 Company: AVIVA, catalog: ARP63725**

QAKAKSKTLDFIDVLLLSKDEDGKKLSDEDIRAEADTFMFEGHDTTASGL

**P4504F2 Company: A.V.I.V.A., catalog: OASG02010** GLWPVAASPWLLLLLVGASWLLAHVLAWTYAFYDNCRRLRCFPQPPRRNWFWGHQG MVNPTEEGMRVLTQLVATYPQGFKV

**GPR75 Company: A.V.I.V.A., catalog: ARP33104**

PSQEESSPCNLQPVNSFGFANSYIAMHYHTTNDLVQEYDSTSAKQIPVPS

**GPR75 Company: AVIVA, catalog: OASG03177**

ALYRNQNYNKLQHVQTRGYTKSPNQLVTPAASRLQLVSAINLSTAKDSKA

**Beta-actin Company: ProteinTech, catalog: 20536-1-AP**
